# A story of viral co-infection, co-transmission and co-feeding in ticks: how to compute an invasion reproduction number

**Published:** 2024-03-22

**Authors:** Giulia Belluccini, Qianying Lin, Bevelynn Williams, Yijun Lou, Zati Vatansever, Martín López-García, Grant Lythe, Thomas Leitner, Ethan Romero-Severson, Carmen Molina-París

**Affiliations:** a T-6, Theoretical Biology and Biophysics, Los Alamos National Laboratory, Los Alamos, 87545, NM, USA; b School of Mathematics, University of Leeds, Leeds, LS2 9JT, UK; c Department of Applied Mathematics, Hong Kong Polytechnic University, Hong Kong SAR, China; d Department of Parasitology, Faculty of Veterinary Medicine, Kafkas University, Kars, Turkey

**Keywords:** co-infection, co-transmission, co-feeding, invasion reproduction number, neutrality, mathematical model, basic reproduction number *PACS:* 02.30.Hq, 87.10.Ed, 87.23.-n, 37N25, 62P10

## Abstract

With a single circulating vector-borne virus, the basic reproduction number incorporates contributions from tick-to-tick (co-feeding), tick-to-host and host-to-tick transmission routes. With two different circulating vector-borne viral strains, resident and invasive, and under the assumption that co-feeding is the only transmission route in a tick population, the invasion reproduction number depends on whether the model system of ordinary differential equations possesses the property of neutrality. We show that a simple model, with two populations of ticks infected with one strain, resident or invasive, and one population of co-infected ticks, does not have Alizon’s neutrality property. We present model alternatives that are capable of representing the invasion potential of a novel strain by including populations of ticks dually infected with the same strain. The invasion reproduction number is analysed with the next-generation method and via numerical simulations.

## 1. Introduction

Co-infection of a single host by at least two distinct viruses provides an opportunity for viruses to exchange genetic information through genomic reassortment or recombination [1, 2]. In fact, entirely novel pathogenic viruses have emerged from reassortment events of less pathogenic parents in nature [3–5]. Co-infection can be thought of as the rate-limiting step in the sudden emergence of genetically distant variants of existing human pathogens such as influenza, SARS-CoV-2, and Crimean Congo Hemorrhagic Fever Virus (CCHFV). Therefore, understanding the dynamics of co-infection in common host species, *e.g.,* arthropods (ticks or mosquitoes), is essential to study the emergence and re-emergence of both new and old human pathogens.

**Figure 1:**
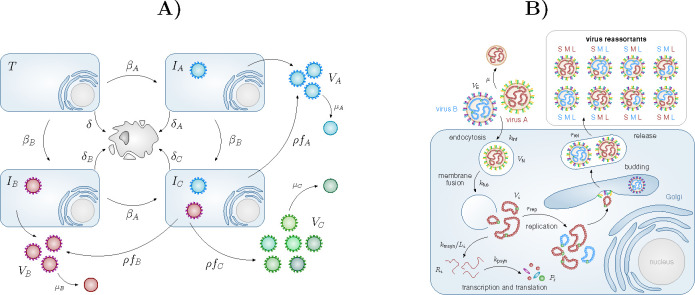
A) If two viral strains, $V_{A}$ and $V_{B}$, are co-circulating, the target cells, T, of an infected host will become singly infected ($I_{A}$ and $I_{B}$), and potentially co-infected ($I_{C}$). Co-infected cells have the potential to generate new viral progeny, different from that of the parental strains: $V_{C}\neq V_{A}$ and $V_{C}\neq V_{B}$. B) A co-infected cell can lead to reassortment events, and produce up to 2^3^ different reassortants.

Genomic reassortment is possible in viruses with segmented genomes, such as the Bunyaviruses, which themselves include lethal pathogens of relevance to public health and of pandemic potential, *e.g.,* Lassa fever, Rift Valley fever and CCHF viruses [6]. Fig. 1 illustrates the dynamics of reassortment at the cellular level for Bunyaviruses, or more generally for a tri-segmented virus. CCHFV is a tick-borne Bunyavirus, with the potential to reassort, and an increasing geographical range due to the changing climate [7, 8]. Understanding how adaptable to different hosts this potentially fatal human pathogen is, what role co-infection (as a first step to genomic reassortment) will play in the generation of potential new viral strains, and how those variants will spread among already infected ticks, is a challenge for theoretical biology.

Due to the ability of ticks to carry multiple viruses or viral strains, epidemiologists have considered co-infection in tick-borne diseases [9–17], superinfection [9], and co-transmission [18–20]. Specifically, epidemiologists are interested in quantifying (or understanding) the invasion potential of a novel virus or strain [21, 22], given the endemic ability of a resident one [23]. In the same way as *R*_0_ provides conditions for successful establishment of a single virus in a susceptible population, the *invasion reproduction number*, *R*_*I*_, lends threshold conditions for successful invasion of a second virus when the population is endemic with the first one [24, 25]. For instance, Gao *et al.* developed a Susceptible-Infected-Susceptible (SIS) model for tick and host populations, and conducted a systematic analysis of invasion by the second virus [18]. More recently, Bushman and Antia have developed a general framework of the interaction between viral strains at the within-host level [26]. Pfab *et al.* have extended the time-since-infection framework of Kermack and McK-endrick [27] for two pathogens [28]. Rovenolt and Tate [29] have developed a model of co-infection to study how within-host interactions between parasites can alter host competition in an epidemic setting. Thao Le *et al.* [30] have studied a two-strain SIS model with co-infection (or co-colonisation) which incorporates variation in transmissibility, duration of carriage, pair-wise susceptibility to co-infection, co-infection duration, and transmission priority effects. Finally, Saad-Roy *et al.* [31] have considered super-infection and its role during the the first stage of an infection on the evolutionary dynamics of the degree to which the host is asymptomatic.

In the case of plant pathogens, recent experimental studies have shown the complex nature of vector-virus-plant interactions and their role in the transmission and replication of viruses as single and co-infections [32]. Allen *et al.* modeled the transmission dynamics of viruses between vectors and plants, under the assumption that co-infection could only take place in plants [24]. Chapwanya *et al.* developed a general deterministic epidemic model of cropvector-borne disease for synergistic co-infection [33]. Miller *et al.* have shown that mathematical models on the kinetics of co-infection of plant cells with two strains could not adequately describe the data [34].

Current mathematical models of co-infection need to be put in perspective, as previously discussed by Lipsitch *et al.* and Alizon [35, 36]. Alizon compared different models of co-infection and raised an issue of *non-neutrality* [36]. He noticed that certain models of co-infection lead to an invasion reproduction number which does not tend to one, in the limit when the invasive and the resident pathogens are the same. To solve this problem, Alizon proposed an alternative model structure, which includes a population of dually infected individuals with the resident pathogen, to achieve the desired neutral invasion reproduction number [36, 37].

In this paper, we first present a mathematical model of a single vector-borne virus to understand the role that different transmission routes play in the dynamics of the infected populations. Then, we study the dynamics of two different viruses, or viral strains, in a tick population making use of a classic co-infection model. After performing an invasion analysis, we explain the issue of non-neutrality of the invasion reproduction number, and propose five neutral alternatives. We conclude the paper with a summary of our alternative proposals, their applicability and limitations.

## 2. Mathematical model of a single viral strain in a population of ticks and their vertebrate hosts

We consider a tick population feeding on a population of vertebrate hosts, where both populations are susceptible to infection with virus $V_{1}$. The host and tick populations are divided into susceptible and infected subsets. In what follows the number of susceptible hosts (ticks) is denoted $n_{0}\left(m_{0}\right)$, and the number of infected hosts (ticks) is denoted $n_{1}\left(m_{1}\right)$, respectively.

The mathematical model considers immigration, death, viral transmission and recovery events in the populations; namely, susceptible hosts and ticks immigrate into the population with rate $\Phi_{H}$ and $\Phi_{T}$, respectively. Susceptible and infected hosts can die with per capita rates $\mu_{0}$ and $\mu_{1}$, respectively, whereas susceptible and infected ticks are characterised by the per capita death rates $\nu_{0}$ and $\nu_{1}$, respectively. We assume an infected host can infect a susceptible tick with rate $\gamma_{1}$, and an infected tick can infect a susceptible host with rate $\beta_{1}$. Both of these transmission events involve a tick feeding on a vertebrate host, and are referred to as *systemic* transmission events [38]. The virus can additionally be transmitted from an infected tick to a susceptible one via co-feeding [39, 40]. This occurs when ticks feed on a host in clusters, and close to each other; that is, on the same host and at the same time. In this instance the virus is transmitted by infected tick saliva, with this route of transmission referred to as *non-systemic* [38]. We denote by $\alpha_{1}$ the rate at which an infected tick can infect a susceptible one via co-feeding. We assume that transmission events follow mass action kinetics. For example, in the case of co-feeding, and with $m_{0}$ and $m_{1}$ the number of susceptible and infected ticks, respectively, the rate of infection for the susceptible population is $\alpha_{1}m_{0}m_{1}$. Finally, once a tick contracts the virus, it remains infected for life [41]. On the other hand, vertebrate hosts are characterised by shortlasting viremia [41, 42]. We, thus, assume that hosts clear the virus with rate $\varphi_{1}$ [43, 44]. The above set of events are brought together in the following system of ordinary differential equations (ODEs), which describe the dynamics of susceptible and infected hosts and ticks:

(1) $$\begin{array}{l} \frac{dn_{0}}{\;dt}\;=\Phi_{H}-\mu_{0}n_{0}-\beta_{1}n_{0}m_{1}+\varphi_{1}n_{1},\\ \frac{dn_{1}}{\;dt}\;=-\mu_{1}n_{1}+\beta_{1}n_{0}m_{1}-\varphi_{1}n_{1},\\ \frac{dm_{0}}{\;dt}\;=\Phi_{T}-\nu_{0}m_{0}-\gamma_{1}m_{0}n_{1}-\alpha_{1}m_{0}m_{1},\\ \frac{dm_{1}}{\;dt}\;=-\nu_{1}m_{1}+\gamma_{1}m_{0}n_{1}+\alpha_{1}m_{0}m_{1}. \end{array}$$

We note that this system of ODEs (1) has a virus-free equilibrium (VFE), $\left(n_{0}^{⋆},0,m_{0}^{⋆},0\right)$, given by

(2) $$n_{0}^{⋆}=\frac{\Phi_{H}}{\mu_{0}},\;m_{0}^{⋆}=\frac{\Phi_{T}}{\nu_{0}}.$$

### 2.1. Basic reproduction number

The basic reproduction number, $R_{0}$, measures the mean number of new infections produced by an infected individual (during its lifetime) in a population at the virus-free equilibrium; that is, when the population is completely susceptible [45]. $R_{0}$ (for the mathematical model (1)) can be calculated making use of the next-generation matrix method [46] as follows. The sub-system of differential equations for ($n_{1},m_{1}$) is linearised at the VFE, and its Jacobian, J, is then written as J≡T+V, with T the 2×2 matrix of transmission events which accounts for new infections in the susceptible population, and V≡J−T, the 2×2 matrix tracking the changes in the state of the infected populations [46]. The next-generation matrix is defined as $K\equiv T{\left(-V\right)}^{-1}$, and the basic reproduction number, $R_{0}$, is given by the largest eigenvalue of K [46]. For our system we have

(3) $$J\equiv \left(\begin{array}{cc} -\mu_{1}-\varphi_{1} & \beta_{1}n_{0}^{⋆}\\ \gamma_{1}m_{0}^{⋆} & -\nu_{1}+\alpha_{1}m_{0}^{⋆} \end{array}\right)$$

with

(4) $$T\equiv \left(\begin{array}{cc} 0 & \beta_{1}n_{0}^{⋆}\\ \gamma_{1}m_{0}^{⋆} & \alpha_{1}m_{0}^{⋆} \end{array}\right),\;\;\text{and}\;\;V\equiv \left(\begin{array}{cc} -\mu_{1}-\varphi_{1} & 0\\ 0 & -\nu_{1} \end{array}\right),$$

so that

(5) $$K\equiv \left(\begin{array}{cc} 0 & \beta_{1}n_{0}^{⋆}/\nu_{1}\\ \gamma_{1}m_{0}^{⋆}/\left(\mu_{1}+\varphi_{1}\right) & \alpha_{1}m_{0}^{⋆}/\nu_{1} \end{array}\right),$$

which in turn implies

(6) $$R_{0}\equiv \frac{1}{2}\left[\frac{\alpha_{1}m_{0}^{⋆}}{\nu_{1}}+\sqrt{{\left(\frac{\alpha_{1}m_{0}^{⋆}}{\nu_{1}}\right)}^{2}+4\frac{\beta_{1}\gamma_{1}m_{0}^{⋆}n_{0}^{⋆}}{\nu_{1}\left(\mu_{1}\;+\;\varphi_{1}\right)}}\right].$$

If $R_{0}<1$, the VFE is stable, and if $R_{0}>1$, it is unstable. The basic reproduction number can be rewritten as

(7) $$R_{0}=\frac{1}{2}\left(R_{TT}+\sqrt{R_{TT}^{2}+4R_{TH}R_{HT}}\right),$$

where we have introduced the following type reproduction numbers [47]

$$R_{TT}=\alpha_{1}\frac{\Phi_{T}}{\nu_{0}\nu_{1}},\;R_{TH}=\beta_{1}\frac{\Phi_{H}}{\mu_{0}\nu_{1}},\;R_{HT}=\gamma_{1}\frac{\Phi_{T}}{\nu_{0}\left(\mu_{1}\;+\;\varphi_{1}\right)},$$

which represent the contribution of each route of transmission, tick-to-tick, tick-to-host and host-to-tick, respectively, to the total number of new infections (of ticks and hosts) in the susceptible population. $R_{TT}$, $R_{TH}$, and $R_{HT}$ correspond to the entries of the next-generation matrix K (see Eq. (5)). $R_{HH}=0$, since the virus cannot be directly transmitted from an infected host to a susceptible one. The expression of the basic reproduction number for a single virus (see Eq. (7)) clearly shows that co-feeding represents a singular route of transmission, compared to systemic routes. For example, $\beta_{1}$ (or $\gamma_{1}$) can be very large, but if $\gamma_{1}$ (or $\beta_{1}$) is negligible, the contribution to $R_{0}$ of viral systemic transmission will be negligible. Therefore, co-feeding events (as characterised by the parameter $\alpha_{1}$), might maintain an epidemic if $R_{TT}>1$. On the other hand, systemic transmission requires both tick-to-host and host-to-tick transmission routes to be non-vanishing, so that there is a chance for $R_{0}>1$, since as soon as either $\beta_{1}$ or $\gamma_{1}$ are equal to zero, $R_{0}=0$ in the absence of co-feeding.

We conclude this section mentioning a novel network approach (developed in Ref. [48]), to compute the parameters $\alpha_{1}$, $\beta_{1}$, and $\gamma_{1}$ from first principles. It is reassuring to note that this approach leads to a next-generation matrix with the same structure as K in Eq. (5).

### 2.2. Parameter values

We make use of recent literature to obtain parameter values for the ODE system (1). Table 1 contains a description of each model parameter, together with its plausible ranges and units. Since infection with CCHFV [42, 49] or *Borrelia* [48] is asymptomatic in ticks and vertebrate hosts (but unfortunately not in humans), we assume it does not affect their death rates; that is, $\mu_{0}=\mu_{1}\equiv \mu$ and $\nu_{0}=\nu_{1}\equiv \nu$ [18]. Given the narrow ranges in Table 1 for $\Phi_{H}$, $\Phi_{T}$, $\varphi_{1}$ and μ, we fix these parameters as follows: $\Phi_{H}=1$ host per day, $\Phi_{T}=2$ ticks per day, $\varphi_{1}=1/6$ per day, and $\mu =10^{-3}$ per day. We derive plausible ranges for the other model parameters making use of Ref. [48], as illustrated in detail in Appendix A.

### 2.3. Visualization of the basic reproduction number

**Figure 2:**
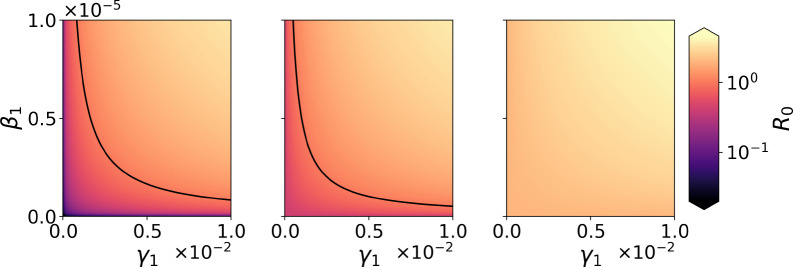
Contribution of $\alpha_{1},\beta_{1}$ and $\gamma_{1}$ to the basic reproduction number, $R_{0}$, given by Eq. (6). Model parameters have been chosen as discussed in Section 2.2 and units for $\alpha_{1}$, $\beta_{1}$ and $\gamma_{1}$ as in Table 1. On the left, $\alpha_{1}=10^{-6}$, in the middle $\alpha_{1}=2\times 10^{-5}$, and on the right $\alpha_{1}=10^{-4}$. The parameters $\gamma_{1}$ and $\beta_{1}$ are varied along the *x*-axis and *y*-axis, respectively, from 0 to their maximum value listed in Table 1. Black curves represent the critical value $R_{0}=1$.

We illustrate the dependence of *R*_0_ on the transmission parameters $\alpha_{1}$, $\beta_{1}$ and $\gamma_{1}$, Fig. 2, making use of (6), and the parameter values from Section 2.2. Lighter colours correspond to greater values of $R_{0}$ (scale on right). Black lines represent a basic reproduction number equal to its critical value of 1. We set $\Phi_{H}$, $\Phi_{T}$, $\varphi_{1}$, and μ to the values specified in Section 2.2, and set $\nu =10^{-2}$ per day (see Appendix A). We consider a different value of *α*_1_ in each panel: on the left, $\alpha_{1}=10^{-6}$, in the middle $\alpha_{1}=2\times 10^{-5}$, and on the right $\alpha_{1}=10^{-4}$ (units as provided in Table 1). The corresponding values of $R_{TT}$ are $R_{TT}=1.2\times 10^{-2}$, $R_{TT}=0.4$, and $R_{TT}=2$. Along the *x*-axis and *y*-axis we vary $\gamma_{1}$ and $\beta_{1}$, respectively, from 0 to their maximum value listed in Table 1. We note that the area under the curve $R_{0}=1$ becomes smaller as $\alpha_{1}$ increases (from left to right), until it becomes zero when co-feeding transmission contributes to make $R_{0}$ greater than one on its own. As one would expect, smaller values of the transmission parameters $\alpha_{1}$, $\beta_{1}$ and $\gamma_{1}$ correspond to lower values of $R_{0}$ (purple regions on the left and middle panels). Finally, we also note the symmetric role of $\beta_{1}$ and $\gamma_{1}$ in $R_{0}$, as shown in (6).

## 3. Two viral strains: tick population and co-feeding transmission

**Figure 3:**
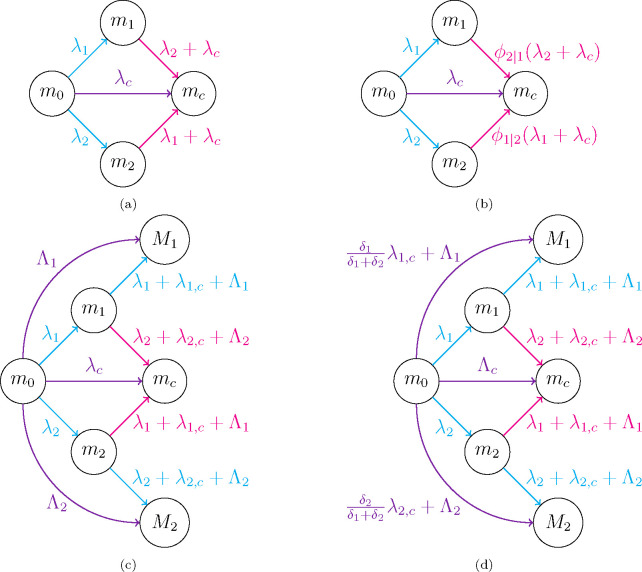
Illustrative diagrams of the mathematical models discussed in the paper for a population of co-feeding ticks with two viral strains. (a) Mathematical model of co-feeding transmission defined by Eq. (8). Transmission rates are defined in Eq. (9). (b) Within-host mathematical model of co-feeding transmission defined by Eq. (12). Transmission rates are defined in Eq. (9). (c) Alizon’s (generalised) proposal for co-infection and co-transmission described in Eq. (13). Transmission rates are defined in Eq. (14). (d) Two-slot mathematical model of co-infection and co-transmission defined in Eq. (15). Transmission rates are defined in Eq. (14) and Eq. (16).

In the previous section, we have shown that co-feeding can sustain an infection among ticks without systemic transmission. The remainder of the paper will focus on the co-feeding route of transmission. We now move to the more complex case where multiple viral strains co-exist, introducing the notions of co-infection and co-transmission. The population of ticks can be infected by two different circulating viral strains, $V_{1}$ and $V_{2}$. $V_{1}$ is considered to be the resident strain and $V_{2}$ the invasive one (*e.g.*, one that emerges once the tick population is endemic with $V_{1}$). The population of ticks can be classified by its infection status in four different compartments, as susceptible and infected ticks with the resident strain, $m_{0}$ and $m_{1}$, respectively, and infected ticks with the invasive strain and co-infected (*i.e.,* infected with both strains $V_{1}$ and $V_{2}$) ticks, $m_{2}$ and $m_{c}$, respectively. Figure 3a shows the mathematical model and the routes of viral transmission considered between different tick compartments. The model corresponds to the following system of ODEs:

(8) $$\begin{array}{l} \frac{dm_{0}}{\;dt}\;=\Phi -\nu_{0}m_{0}-m_{0}\left(\lambda_{1}+\lambda_{2}+\lambda_{c}\right),\\ \frac{dm_{1}}{\;dt}\;=-\nu_{1}m_{1}+m_{0}\lambda_{1}-m_{1}\left(\lambda_{2}+\lambda_{c}\right),\\ \frac{dm_{2}}{\;dt}\;=-\nu_{2}m_{2}+m_{0}\lambda_{2}-m_{2}\left(\lambda_{1}+\lambda_{c}\right),\\ \frac{dm_{c}}{\;dt}\;=-\nu_{c}m_{c}+m_{0}\lambda_{c}+m_{1}\left(\lambda_{2}+\lambda_{c}\right)+m_{2}\left(\lambda_{1}+\lambda_{c}\right), \end{array}$$

where we have introduced

(9) $$\begin{array}{l} \lambda_{1}=\alpha_{1}m_{1}+\delta_{1}\left(1-\epsilon_{c}\right)m_{c},\\ \lambda_{2}=\alpha_{2}m_{2}+\delta_{2}\left(1-\epsilon_{c}\right)m_{c},\\ \lambda_{c}=\left(\delta_{1}+\delta_{2}\right)\epsilon_{c}m_{c}, \end{array}$$

with $\epsilon_{c}\in [0,\;1]$ representing the probability of co-transmission ($V_{1}$ and $V_{2}$). We have assumed that the $m_{1}\left(m_{2}\right)$ population has transmission parameter $\alpha_{1}\left(\alpha_{2}\right)$ for $V_{1}\left(V_{2}\right)$, and the $m_{c}$ (co-infected) population has transmission parameter $\delta_{1}$ for $V_{1}$ and $\delta_{2}$ for $V_{2}$, respectively. We have also slightly abused notation by writing $\Phi_{T}=\Phi$.

### 3.1. Basic reproduction number

The mathematical model defined by the system of ODEs (8) has a virus-free equilibrium (VFE), $\left(m_{0}^{⋆},0,0,0\right)$, with $m_{0}^{⋆}=\frac{\Phi }{\nu_{0}}$. To compute its basic reproduction number, we make use of the next-generation matrix method, as illustrated in detail in Section 2.1. The T and V matrices are given by

$$T=\left(\begin{array}{ccc} \alpha_{1}m_{0}^{⋆} & 0 & \delta_{1}\left(1-\epsilon_{c}\right)m_{0}^{⋆}\\ 0 & \alpha_{2}m_{0}^{⋆} & \delta_{2}\left(1-\epsilon_{c}\right)m_{0}^{⋆}\\ 0 & 0 & \left(\delta_{1}+\delta_{2}\right)\epsilon_{c}m_{0}^{⋆} \end{array}\right),\;and\;\;V=\left(\begin{array}{ccc} -\nu_{1} & 0 & 0\\ 0 & -\nu_{2} & 0\\ 0 & 0 & -\nu_{c} \end{array}\right).$$

Thus, by computing the eigenvalues of the next-generation matrix, $K=T{\left(-V\right)}^{-1}$, the basic reproduction number of system (8) can be shown to be $R_{0}=max\left\{R_{1},R_{2},R_{c}\right\}$, with

$$R_{1}=\frac{\alpha_{1}}{\nu_{1}}m_{0}^{⋆},\;R_{2}=\frac{\alpha_{2}}{\nu_{2}}m_{0}^{⋆},\;R_{c}=\frac{\left(\delta_{1}\;+\;\delta_{2}\right)\epsilon_{c}}{\nu_{c}}m_{0}^{⋆}.$$

Following the results from Ref. [18, Proposition 2.1], we can explore the boundary equilibria of system (8):

1. The virus-free equilibrium, $E_{0}=\left(m_{0}^{⋆},0,\;0,\;0\right)$, always exists.
2. The endemic equilibrium with $V_{1},E_{1}=\left(\frac{\nu_{1}}{\alpha_{1}},(1-\frac{1}{R_{1}})\frac{\Phi }{\nu_{1}},0,\;0\right)$, exists if and only if $R_{1}>1$.
3. The endemic equilibrium with $V_{2},E_{2}=\left(\frac{\nu_{2}}{\alpha_{2}},0,(1-\frac{1}{R_{2}})\frac{\Phi }{\nu_{2}},0\right)$, exists if and only if $R_{2}>1$.
4. The endemic equilibrium with co-infected ticks, $E_{c}=\left(\frac{\nu_{c}}{(\delta_{1}+\delta_{2})\epsilon_{c}},0,\;0,(1-\frac{1}{R_{c}})\frac{\Phi }{\nu_{c}}\right)$, exists if and only if $\epsilon_{c}=1$, and $R_{c}>1$.

### 3.2. Invasion reproduction number

We now assume that $R_{1}>1$, so that the endemic equilibrium $E_{1}$ of (8) exists. We write

$${\bar{m}}_{0}=\frac{\nu_{1}}{\alpha_{1}},\;{\bar{m}}_{1}=\left(1-\frac{1}{R_{1}}\right)\frac{\Phi }{\nu_{1}},$$

with $E_{1}=\left({\bar{m}}_{0},{\bar{m}}_{1},0,\;0\right)$. We aim to calculate the invasion reproduction number of $V_{2}$ by means of the next-generation matrix method. To this end, we identify the invasive sub-system of $V_{2}$ of Eq. (8), linearise it around $E_{1}$, compute its Jacobian matrix, and define the 2 × 2 matrices T and V. We can write

$$T\equiv \left(\begin{array}{cc} \alpha_{2}{\bar{m}}_{0} & \delta_{2}\left(1-\epsilon_{c}\right){\bar{m}}_{0}\\ \alpha_{2}{\bar{m}}_{1} & \begin{array}{c} \left.\delta_{1}+\delta_{2}\right)\epsilon_{c}\left({\bar{m}}_{0}+{\bar{m}}_{1}\right)\\ +\delta_{2}\left(1-\epsilon_{c}\right){\bar{m}}_{1} \end{array} \end{array}\right),\;\;\text{and}\;\;V\equiv \left(\begin{array}{cc} -\alpha_{1}{\bar{m}}_{1}-\nu_{2} & 0\\ \alpha_{1}{\bar{m}}_{1} & -\nu_{c} \end{array}\right).$$

The next-generation matrix, $K=T{\left(-V\right)}^{-1}$, is given by

$$K\equiv \left(\begin{array}{ll} R_{22} & R_{c2}\\ R_{2c} & R_{cc} \end{array}\right),$$

with the type reproduction numbers $R_{22},R_{2c},R_{c2}$, and $R_{cc}$ given by

$$\begin{array}{l} R_{22}\;=\frac{\alpha_{2}{\bar{m}}_{0}}{\alpha_{1}{\bar{m}}_{1}\;+\;\nu_{2}}\;+\;\frac{\alpha_{1}{\bar{m}}_{1}}{\alpha_{1}{\bar{m}}_{1}\;+\;\nu_{2}}\frac{\delta_{2}\left(1\;-\;\epsilon_{c}\right){\bar{m}}_{0}}{\nu_{c}},\\ R_{c2}\;=\frac{\delta_{2}\;\left(1\;-\;\epsilon_{c}\right){\bar{m}}_{0}}{\nu_{c}},\\ R_{2c}\;=\frac{\alpha_{2}{\bar{m}}_{1}}{\alpha_{1}{\bar{m}}_{1}\;+\;\nu_{2}}\;+\;\frac{\alpha_{1}{\bar{m}}_{1}}{\alpha_{1}{\bar{m}}_{1}\;+\;\nu_{2}}\frac{\left(\delta_{1}\;+\;\delta_{2}\right)\;\epsilon_{c}\;\left({\bar{m}}_{0}\;+\;{\bar{m}}_{1}\right)\;+\;\delta_{2}\;\left(1\;-\;\epsilon_{c}\right){\bar{m}}_{1}}{\nu_{c}},\\ R_{cc}\;=\frac{\left(\delta_{1}\;\;+\;\;\delta_{2}\right)\;\epsilon_{c}\;\left({\bar{m}}_{0}\;+\;{\bar{m}}_{1}\right)\;+\;\;\delta_{2}\;\left(1\;-\;\epsilon_{c}\right){\bar{m}}_{1}}{\nu_{c}}. \end{array}$$

The eigenvalues of K are solutions of the following quadratic equation

$$\lambda^{2}-\left(R_{22}+R_{cc}\right)\lambda +\left(R_{22}R_{cc}-R_{c2}R_{2c}\right)=0.$$

The invasion reproduction number, $R_{I}$, is the largest eigenvalue of K, *i.e.,*

(10) $$R_{I}=\frac{\left(R_{22}\;+\;R_{cc}\right)\;+\;\sqrt{{\left(R_{22}\;+\;R_{cc}\right)}^{2}\;-\;4\;\left(R_{22}\;R_{cc}\;-\;R_{c2}\;R_{2c}\right)}}{2}.$$

When $R_{I}>1$, that is, $R_{22}\;+\;R_{cc}-R_{22}\;R_{cc}\;+\;R_{c2}\;R_{2c}>1$, $V_{2}$ is able to invade a tick population where the resident strain *V*_1_ is endemic.

## 4. Alternative neutral models of co-feeding, co-infection, and co-transmission

The invasion reproduction number of the mathematical model from Section 3.2 is not *neutral* [23, 37]. By neutrality, we mean the following: in the limit when the invasive strain tends to the resident one, there should be no advantage for either strain, and thus, $R_{I}\to 1$. One can show for $R_{I}$ given by Eq. (10) that RI→1. In fact, we have $R_{I}\to 1$ iff $\delta_{2}=\frac{\alpha_{1}}{1+R_{1}}$, and $R_{I}\to 1$ iff $\delta_{2}+\delta_{1}=\frac{\alpha_{1}}{R_{1}}$, for $\epsilon_{c}=0$ and $\epsilon_{c}=1$, respectively, under the assumption that infection does not affect the death rate, i.e., $\nu_{0}=\nu_{1}=\nu_{2}=\nu_{c}$. The issue of neutrality in co-infection models was brought up by Samuel Alizon in Ref. [37] and Lipsitch *et al.* in Ref. [35]. We now present five alternative *neutral* formulations of the previous model. The first (and less optimal) option for obtaining a neutral model is to force $R_{I}\to 1$ and in turn, consider the constraints this condition imposes on some of the model parameters. The second one, as proposed by us to Samuel Alizon in private communication, is to consider a normalised invasion reproduction number; that is, define $R_{I}^{N}=\frac{R_{I}}{{lim}_{2\to 1}\;R_{I}}$, where by ${lim}_{2\to 1}\;R_{I}$, we mean the value of the invasion reproduction number in the limit when the invasive strain tends to the resident one (see Section 4.1). The third one generalises the mathematical model (8) by introducing the idea of within-host probability of invasion (see Section 4.2). The fourth one, as proposed by Alizon [37], is to consider a more general class of models, with doubly infected individuals (see Section 4.3). A final one that we propose in Section 4.4, is a generalisation of the approach of Alizon [23, 37], which clearly articulates the issue of co-transmission.

### 4.1. A normalised invasion reproduction number

The invasion reproduction number given by Eq. (10) is not neutral. Let us then define a *normalised* invasion reproduction number, $R_{I}^{N}$, as follows

(11) $$R_{I}^{N}=\frac{R_{I}}{\;{lim}_{2\to 1}\;R_{I}},$$

where ${lim}_{2\to 1}\;$ means $\nu_{2}\to \nu_{1}$, $\alpha_{2}\to \alpha_{1}$, and $\delta_{2}\to \delta_{1}$ for our co-infection model (see Eq. (8)). So defined, it is clear that ${lim}_{2\to 1}\;R_{I}^{N}=1$, which is the desired neutrality condition. We note that the condition for the invasive strain to have the potential to become established is $R_{I}>1$. Now that we have introduced a normalised invasion reproduction number, this condition becomes $R_{I}^{N}>{\left({lim}_{2\to 1}\;R_{I}\right)}^{-1}$.

### 4.2. A model with within-host invasion

The fitness advantage of the invasive strain in model (8) stems from the assumption that $V_{2}$ can infect susceptible ticks and infected ticks by the resident strain with the same rate. However, this may not be realistic. For instance, a small amount of transmitted (invasive) virus may be less likely to establish infection in a tick that already has a high resident viral load, compared to a fully susceptible tick. The probability of within-host invasion will, thus, depend on the relative within-host fitnesses of the invasive and resident strains. Therefore, we can adapt the previous model (Eq. (8)) by introducing the parameter $\phi_{i|j}$, which is the probability that strain i can establish co-infection in a tick already infected by strain *j*, given that there is transmission of strain i via co-feeding. This is similar to the super-infection framework described by Alizon in Ref. [36]. The model can then be described by the following system of ODEs:

(12) $$\begin{array}{l} \frac{dm_{0}}{\;dt}\;=\Phi -\nu_{0}m_{0}-m_{0}\left(\lambda_{1}\;+\;\lambda_{2}\;+\;\lambda_{c}\right),\\ \frac{dm_{1}}{\;dt}\;=-\nu_{1}m_{1}\;+\;m_{0}\lambda_{1}-m_{1}\phi_{2|1}\left(\lambda_{2}\;+\;\lambda_{c}\right),\\ \frac{dm_{2}}{\;dt}\;=-\nu_{2}m_{2}\;+\;m_{0}\lambda_{2}-m_{2}\phi_{1|2}\left(\lambda_{1}\;+\;\lambda_{c}\right),\\ \frac{dm_{c}}{\;dt}\;=-\nu_{c}m_{c}\;+\;m_{0}\lambda_{c}\;+\;m_{1}\phi_{2|1}\left(\lambda_{2}\;+\;\lambda_{c}\right)\;+\;m_{2}\phi_{1|2}\left(\lambda_{1}\;+\;\lambda_{c}\right), \end{array}$$

where $\lambda_{1}$, $\lambda_{2}$ and $\lambda_{c}$ are defined by Eq. (9). The transmission events of this model are summarised in Fig. 3b. In Appendix B we show that the invasion reproduction number for this model satisfies the desired neutrality condition.

### 4.3. A generalisation of Alizon’s proposal

The mathematical model proposed by Alizon in Ref. [37] to obtain a neutral invasion reproduction number requires two additional populations (see Fig. 3c), namely the populations of doubly infected ticks with either $V_{1}$ or $V_{2}$, denoted by $M_{1}$ and $M_{2}$, respectively. Thus, there are six different tick compartments: $m_{0}$, susceptible ticks, $m_{1}$, $m_{2}$, ticks (singly) infected with either $V_{1}$ or $V_{2}$, $M_{1}$, $M_{2}$, doubly infected ticks with either $V_{1}$ or $V_{2}$, and $m_{c}$, co-infected ticks with both $V_{1}$ and $V_{2}$. We note that the co-infection models in Ref. [23] do not consider co-transmission, but it is discussed in Ref. [37]. Thus, in what follows, and to develop a mathematical model of co-infection and co-transmission in co-feeding ticks, we explain in detail what happens when a co-infected tick transmits virus to a singly infected tick. If co-transmission of both, resident and invasive, strains occurs, the singly infected tick can only acquire one new viral strain, since the mathematical model does not accommodate triply infected ticks. Therefore, if co-transmission takes place, a singly infected tick will acquire $V_{1}$ with probability $\frac{\delta_{1}}{\delta_{1}+\delta_{2}}$, or $V_{2}$ with probability $\frac{\delta_{2}}{\delta_{1}+\delta_{2}}$. Hence, the overall rate at which a co-infected tick transmits $V_{1}$ to a singly infected tick is $\left(1-\epsilon_{c}\right)\delta_{1}+\left(\delta_{1}+\delta_{2}\right)\epsilon_{c}\frac{\delta_{1}}{\delta_{1}+\delta_{2}}=\delta_{1}$, where the first term represents transmission of $V_{1}$ if no co-transmission, and the second term represents transmission of $V_{1}$ in the event of co-transmission. Similarly, the overall rate a co-infected tick transmits $V_{2}$ to a singly infected tick is $\delta_{2}$. We now write down the system of ODEs for Alizon’s generalised mathematical model of a co-feeding tick population, with two circulating viral strains, which allows for co-infection and co-tranmission, and at most doubly infected ticks (with the same strain $M_{1}$ and $M_{2}$, or with different ones $m_{c}$). We have

(13) $$\begin{array}{l} \frac{dm_{0}}{\;dt}\;=\Phi -\nu_{0}m_{0}-m_{0}\left(\lambda_{1}+\lambda_{2}+\lambda_{1,c}+\lambda_{2,c}+\Lambda_{1}+\Lambda_{2}\right),\\ \frac{dm_{1}}{\;dt}\;=-\nu_{1}m_{1}+m_{0}\lambda_{1}-m_{1}\left(\lambda_{1}+\lambda_{2}+\lambda_{1,c}+\lambda_{2,c}+\Lambda_{1}+\Lambda_{2}\right),\\ \frac{dm_{2}}{\;dt}\;=-\nu_{2}m_{2}+m_{0}\lambda_{2}-m_{2}\left(\lambda_{1}+\lambda_{2}+\lambda_{1,c}+\lambda_{2,c}+\Lambda_{1}+\Lambda_{2}\right),\\ \frac{dm_{c}}{\;dt}\;=-\nu_{c}m_{c}+m_{0}\left(\lambda_{1,c}+\lambda_{2,c}\right)+m_{1}\left(\lambda_{2}+\lambda_{2,c}+\Lambda_{2}\right)+m_{2}\left(\lambda_{1}+\lambda_{1,c}+\Lambda_{1}\right),\\ \frac{dM_{1}}{\;dt}\;=-v_{1}M_{1}+m_{0}\Lambda_{1}+m_{1}\left(\lambda_{1}+\lambda_{1,c}+\Lambda_{1}\right),\\ \frac{dM_{2}}{\;dt}\;=-v_{2}M_{2}+m_{0}\Lambda_{2}+m_{2}\left(\lambda_{2}+\lambda_{2,c}+\Lambda_{2}\right), \end{array}$$

where we define

(14) $$\begin{array}{l} \lambda_{1}\;=\alpha_{1}m_{1}+\delta_{1}\left(1-\epsilon_{c}\right)m_{c}+2\kappa_{1}\left(1-\epsilon_{1}\right)M_{1},\\ \lambda_{2}\;=\alpha_{2}m_{2}+\delta_{2}\left(1-\epsilon_{c}\right)m_{c}+2\kappa_{2}\left(1-\epsilon_{2}\right)M_{2},\\ \lambda_{1,c}\;=\delta_{1}\epsilon_{c}m_{c},\\ \lambda_{2,c}\;=\delta_{2}\epsilon_{c}m_{c},\\ \Lambda_{1}\;=2\kappa_{1}\epsilon_{1}M_{1},\\ \Lambda_{2}\;=2\kappa_{2}\epsilon_{2}M_{2}, \end{array}$$

with $\epsilon_{c}$, the probability of co-transmission of the two viral strains by a co-infected $\left(m_{c}\right)$ tick, and with $\epsilon_{1}\left(\epsilon_{2}\right)$ the probability of co-transmission by a doubly infected tick $M_{1}\left(M_{2}\right)$, respectively. The original model of co-infection with co-transmission defined in Ref. 37] assumed $\epsilon_{1}=\epsilon_{2}=\epsilon_{c}=\epsilon$. We warn the reader that we have defined $\lambda_{1}$ and $\lambda_{2}$ to mean two different things in Eq. (14) and Eq. (9). We shall always clarify in what follows, which of the two definitions is implied. Fig. 3c shows the transmission events described by Eq. (13). We note that co-transmission by the $M_{1}$ tick population to a susceptible tick implies double transmission of the resident viral strain $V_{1}$, and that co-transmission by the $M_{2}$ tick population (to a susceptible tick) implies double transmission of the resident strain $V_{2}$. Finally, the parameter $\kappa_{1}\left(\kappa_{2}\right)$ is the rate of transmission of a single copy of $V_{1}\left(V_{2}\right)$ from an $M_{1}\left(M_{2}\right)$ tick to a susceptible one; thus, the factor of 2 in the previous expression for $\Lambda_{1}\left(\Lambda_{2}\right)$. We remind the reader that the model defined above includes death, immigration and transmission events. We have assumed each tick compartment has a different death rate, and immigration replenishes the susceptible tick compartment. In Appendix C we carefully derive the invasion reproduction number of this model and show its neutrality.

### 4.4. Two-slot model of co-feeding, co-infection and co-transmission

In the model defined by Eq. (13), a co-transmission event from a co-infected tick, in the $m_{c}$ compartment, to a susceptible tick implies the transmission of both viral strains at once. Here we extend the previous model to allow for the possibility that such a co-transmission event could instead result in the transmission of two copies of $V_{1}$ or two copies of $V_{2}$. The idea of this generalised *two-slot model* is as follows: since ticks can be at most doubly infected, we assume each tick has two *infection slots* that can be occupied (or not). In the previous model, “co-transmission” to a susceptible tick meant transmission of both viral strains. In this model “co-transmission” means occupying both slots, in such a way, that the slots can be occupied by two copies of the same virus (leading to $M_{1}$ or $M_{2}$ ticks), or two different strains (leading to $m_{c}$ ticks). The dynamics of the two-slot model can be written as

(15) $$\begin{array}{l} \frac{dm_{0}}{\;dt}\;=\Phi -\nu_{0}m_{0}-m_{0}\left(\lambda_{1}+\lambda_{2}+\lambda_{1,c}+\lambda_{2,c}+\Lambda_{1}+\Lambda_{2}\right),\\ \frac{dm_{1}}{\;dt}\;=-\nu_{1}m_{1}+m_{0}\lambda_{1}-m_{1}\left(\lambda_{1}+\lambda_{2}+\lambda_{1,c}+\lambda_{2,c}+\Lambda_{1}+\Lambda_{2}\right),\\ \frac{dm_{2}}{\;dt}\;=-\nu_{2}m_{2}+m_{0}\lambda_{2}-m_{2}\left(\lambda_{1}+\lambda_{2}+\lambda_{1,c}+\lambda_{2,c}+\Lambda_{1}+\Lambda_{2}\right),\\ \frac{dm_{c}}{\;dt}\;=-\nu_{c}m_{c}+m_{0}\Lambda_{c}+m_{1}\left(\lambda_{2}+\lambda_{2,c}+\Lambda_{2}\right)+m_{2}\left(\lambda_{1}+\lambda_{1,c}+\Lambda_{1}\right),\\ \frac{dM_{1}}{\;dt}\;=-v_{1}M_{1}+m_{0}\left(\Lambda_{1}+\frac{\delta_{1}}{\delta_{1}\;+\;\delta_{2}}\lambda_{1,c}\right)+m_{1}\left(\lambda_{1}+\lambda_{1,c}+\Lambda_{1}\right),\\ \frac{dM_{2}}{\;dt}\;=-v_{2}M_{2}+m_{0}\left(\Lambda_{2}+\frac{\delta_{2}}{\delta_{1}\;+\;\delta_{2}}\lambda_{2,c}\right)+m_{2}\left(\lambda_{2}+\lambda_{2,c}+\Lambda_{2}\right), \end{array}$$

where $\lambda_{1}$, $\lambda_{2}$, $\lambda_{1,c}$, $\lambda_{1,c}$, $\Lambda_{1}$, and $\Lambda_{2}$ have been defined in Eq. (13), and with $\Lambda_{c}$ given by

(16) $$\Lambda_{c}=\frac{2\delta_{1}\delta_{2}}{\delta_{1}\;+\;\delta_{2}}\epsilon_{c}m_{c}.$$

In Appendix D we describe in great detail the transmission events considered in the two-slot mathematical model, show the existence of an endemic equilibrium for $V_{1}$, and prove that the model leads to a neutral invasion reproduction number.

### 4.5. Numerical study of the invasion reproduction number

In Section 3 we have defined and computed the invasion reproduction number for a mathematical model of co-infection and co-transmission in co-feeding ticks. We have argued that such a model is not neutral, and have in turn proposed different mathematical models which do not suffer from such problem. We now propose a numerical study of the invasion reproduction number for the “not-neutral” model introduced in Section 3, as well as the invasion reproduction number for the model solutions proposed above to guarantee neutrality.

In what follows we assume that κ1=α12, and κ2=α22, which are appropriate choices when considering viral infections or micro-parasites [37]. As discussed by Alizon in Ref. [37], if an infected host (by a certain viral strain) is re-infected by the exact same strain, we do not expect to see a change in its viral load (and hence in transmission rate). For co-infected ticks in the $m_{c}$ compartment (and infected by both $V_{1}$ and $V_{2}$), it is reasonable to hypothesise that potential within-tick interactions between the two strains do not lead to a change in transmission rates, when compared to doubly infected ticks in the $M_{1}$ or $M_{2}$ compartments. Thus, we assume $\delta_{1}=\kappa_{1}$ and $\delta_{2}=\kappa_{2}$. Finally, and as justified earlier, we set $\nu_{0}=\nu_{1}=\nu_{2}=\nu_{c}=v_{1}=v_{2}=10^{-2}$ per day. We also fix the immigration rate to be Φ=2 ticks per day, and $\alpha_{1}=10^{-4}$ per tick per day. These choices lead to a basic reproduction number of $R_{1}=2$ for the resident strain, $V_{1}$.

**Figure 4:**
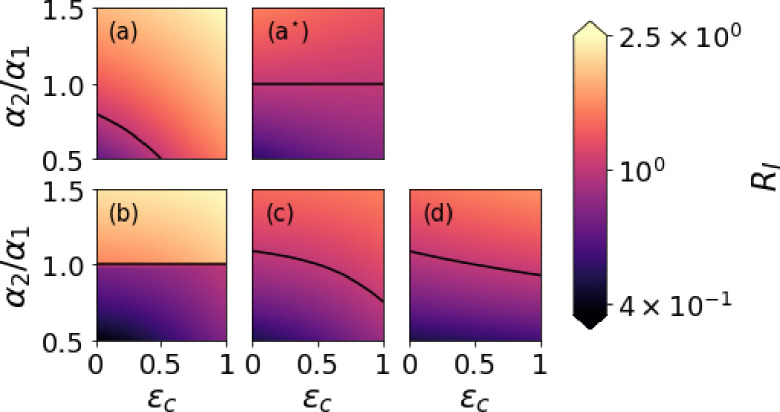
Heatmaps of the invasion reproduction number for (a) the “not-neutral” model (10), ($a^{⋆}$) the normalised proposal (11), (b) the within-host model with $\phi_{i|j}$, Eq. (12), (c) Alizon’s model with co-transmission (13), and (d) the two-slot mathematical model (15). The *x*-axis represents $\epsilon_{c}$, the probability of co-transmission from co-infected ticks. The *y*-axis shows the ratio $\alpha_{2}/\alpha_{1}$ in the range [0.5, 1.5]. We set $\alpha_{1}=10^{-4}$ per tick per day. Black lines mark the contours where the invasion reproduction number, $R_{I}$, is equal to 1. For Alizon’s model and the two-slot extension, we have set $\epsilon_{1}=\epsilon_{2}=1/2$.

In Fig. 4 we compare how different values of the transmission parameter, $\alpha_{2}$, and the co-transmission probability, $\epsilon_{c}$, affect the invasion reproduction number, $R_{I}$, computed in the “not-neutral” scenario (10) (panel (a)), in the normalized proposal of Eq. (11) (panel ($a^{⋆}$)), in the within-host model (12) (panel (b)), in Alizon’s model with co-transmission (13) (panel (c)), and in the two-slot mathematical model (15) (panel (d)). In particular, $\epsilon_{c}$ is varied along the *x*-axis, whereas the ratio α2α1 is varied from 0.5 to 1.5 along the *y*-axis. Black lines mark the contours where the invasion reproduction number, $R_{I}$, is equal to 1. For Alizon’s model and the two-slot extension, we have set ϵ1=ϵ2=12. For the within-host model, we define the probability that strain i can establish co-infection in a tick already infected by strain j as follows

$$\phi_{i|j}=\left\{\begin{array}{ll} 1, & \;\text{if}\;\;\alpha_{i}>\alpha_{j},\\ 0, & \;\text{if}\;\;\alpha_{i}\leq \alpha_{j}. \end{array}\right.$$

We note that the highest values of the invasion reproduction number occur in panel (a) and (b) of Fig. 4. In panel (a), the invasion reproduction number is clearly not neutral, since $R_{I}>1$ when $\alpha_{2}=\alpha_{1}$, and also for some regions of parameter space with $\alpha_{2}<\alpha_{1}$. For this model, if infected ticks with the invasive strain, $V_{2}$, are rare compared to ticks infected with the resident strain, $V_{1}$, then $V_{2}$ has an initial advantage over $V_{1}$. Each tick infected with the invasive strain has the opportunity to infect a much larger number of ticks (${\bar{m}}_{0}+{\bar{m}}_{1}$), than those which can be infected by a tick from the $m_{1}$ compartment. This allows $V_{2}$ to invade the $V_{1}$ endemic system, for large enough values of $\alpha_{2}$ and $\epsilon_{c}$. The co-transmission probability, $\epsilon_{c}$, affects the value of $R_{I}$, since it changes the rate at which co-infected ticks transmit $V_{1}$ and $V_{2}$ to susceptible ticks, $m_{0}$. These rates are $\delta_{1}+\epsilon_{c}\delta_{2}$ and $\delta_{2}+\epsilon_{c}\delta_{1}$, respectively. Therefore a higher probability of co-transmission enables both strains to be transmitted more often. For the normalised invasion reproduction number in panel ($a^{⋆}$), $R_{I}=1$ when $\alpha_{2}=\alpha_{1}$, for every value of $\epsilon_{c}$, given its definition. When $\alpha_{2}\neq \alpha_{1}$, $R_{I}$ does depend on $\epsilon_{c}$, but less so than for the model of panel (a), since ${lim}_{2\to 1}\;R_{I}$ increases with $\epsilon_{c}$. In panel (c), showing the invasion reproduction number for Alizon's model, when ϵc=12 (equal to $\epsilon_{1}$ and $\epsilon_{2}$), $R_{I}=1$ for $\alpha_{2}=\alpha_{1}$. As $\epsilon_{c}$ increases, so does the value of $R_{I}$, since a higher co-transmission probability enables $V_{2}$ to be transmitted along with $V_{1}$ more often. The invasion reproduction number of the two-slot model in panel (d) behaves in a similar fashion. However, increasing $\epsilon_{c}$ does not give as much of an advantage to the invasive strain, since co-transmission events can result in the transmission of two copies of $V_{1}$.

## 5. Discussion and conclusions

In this paper, we consider the role of different transmission routes for a single vector-borne virus in a population of ticks and vertebrate hosts. We then study co-infection and co-transmission of two circulating vector-borne viral strains in a population of co-feeding ticks. We define and compute both the basic reproduction number and the invasion reproduction number, which provides the conditions under which a new variant can emerge (possibly endogenously from genomic reassortment). We illustrate how a classic and *intuitive* model of invasion was not, in fact, neutral with respect to the invading strain; that is, using this model to understand, for example, the minimum selective advantage that needs to be present for a invading strain to take hold of an endemic population (with the resident one) will privilege one strain over the other. This is not a problem *per se*, as it might be the correct model from a mechanistic perspective. However, it is important to characterise the underlying properties of a mathematical model, especially if it is intended to be used as part of an inference procedure. We also presented several alternative formulations of co-infection and co-transmission models that are, by definition neutral. We have shown that each model has distinct and specific behaviour concerning the invasion reproduction number. The take-home message of this review is that the assumptions used to model these important and complex infection systems matter, specially when making inferences about pathogens of potential pandemic emergence. In the real world, the choice of model, from the different alternatives presented and discussed here, will clearly depend on the virus, as well as the immunology and ecology of the hosts those viruses infect.

In conclusion, we note that while we have focused on deterministic models of tick-borne disease transmission, stochastic analogous may be considered instead, particularly when studying the invasion potential of a rare circulating viral strain [15, 51, 52]. In a stochastic framework, the reproduction number is defined as a random variable rather than as an average [53], since its distribution encodes the probability of an epidemic occurring if a pathogen is introduced into a fully susceptible population by a small number of infected individuals [53]. Thus, future work should include a study of the invasion reproduction number probability distribution, as well as an exploration of the issue of non-neutrality making use of stochastic approaches [54]. Finally, given recent reports which indicate an increase in the number of Zika and Dengue virus co-infection cases in expanding co-endemic regions [55], it is of utmost importance to have suitable within-host mathematical models to study the impact of co-infection on viral infection dynamics.

## Figures and Tables

**Table 1:** Model parameters introduced in (1) (top half), and (8), (12), (13), and (15) (bottom half).

Parameter	Event	Range	Units	Reference

$\beta_{1}$	$H_{0}+T_{1}\to H_{1}+T_{1}$	[10^−7^, 10^−5^]	1/day/tick	[48]
$\gamma_{1}$	$T_{0}+H_{1}\to T_{1}+H_{1}$	[10^−5^, 10^−2^]	1/day/host	[48]
$\alpha_{1}$	$T_{0}+T_{1}\to T_{1}+T_{1}$	[10^−6^, 10^−4^]	1/day/tick	[48]
$\nu_{0}$	Death rate of $T_{0}$	10^−2^	1/day	[48]
$\nu_{1}$	Death rate of $T_{1}$	10^−2^	1/day	[48]
$\mu_{0}$	Death rate of $H_{0}$	[2.8 × 10^−4^, 2.8 × 10^−3^]	1/day	[50]
$\mu_{1}$	Death rate of $H_{1}$	[2.8 × 10^−4^, 2.8 × 10^−3^]	1/day	[50]
$\Phi_{T}$	Arrival of ticks	[0.5, 3.5]	tick/day	[38]
$\Phi_{H}$	Arrival of hosts	[0.5, 1.5]	host/day	[38]
$\varphi_{1}$	$H_{1}\to H_{0}$	[1/7,1/5]	1/day	[44]

$\alpha_{2}$	$T_{0}+T_{2}\to T_{2}+T_{2}$	[10^−6^, 10^−4^]	1/day/tick	[48]
$\delta_{1}$	Transmission of $V_{1}$ by $T_{c}$	[10^−6^, 10^−4^]	1/day/tick	Assumed
$\delta_{2}$	Transmission of $V_{2}$ by $T_{c}$	[10^−6^, 10^−4^]	1/day/tick	Assumed
$\kappa_{1}$	Transmission of one copy of $V_{1}$ from $M_{1}$	[10^−6^, 10^−4^]	1/day/tick	Assumed
$\kappa_{2}$	Transmission of one copy of $V_{2}$ from $M_{2}$	[10^−6^, 10^−4^]	1/day/tick	Assumed
$\epsilon_{c}$	Probability of co-transmission	[0, 1]	-	-
$\epsilon_{1}$	Probability of dual transmission of $V_{1}$	[0, 1]		-
$\epsilon_{2}$	Probability of dual transmission of $V_{2}$	[0,1]		-
$\nu_{2}$	Death rate of $T_{2}$	10^−2^	1/day	[48]
$\nu_{c}$	Death rate of $T_{c}$	10^−2^	1/day	[48]

## Data Availability

Numerical codes (Python) to reproduce Figure 2 and Figure 4, as well as the Mathematica notebook to reproduce proofs and results from Appendix D, are deposited at https://github.com/MolEvolEpid/coinfection_cotransmission_cofeeding_in_ticks.

## Highlights

- We introduce a mathematical model of a single vector-borne virus in a population of ticks and hosts, with three different transmission routes, and derive its basic reproduction number.
- We study the dynamics of two different co-circulating viruses, or viral strains, in a tick population making use of a classic co-infection model.
- After performing an invasion analysis, we compute the invasion reproduction number, explain the issue of its non-neutrality, and propose five neutral alternatives.
- We conclude the paper with a summary of our proposals, their applicability and limitations.

## Appendix A. Parameters from network approach

Since CCHFV infection is asymptomatic in ticks and vertebrate hosts, we assume that it does not affect their death rates; that is, $\mu_{0}=\mu_{1}\equiv \mu$ and $\nu_{0}=\nu_{1}\equiv \nu$. Moreover, for the purposes of this section, we fix the values of some parameters within the ranges in Table 1 as follows: $\Phi_{H}=1$ host per day, $\Phi_{T}=2$ ticks per day, $\varphi_{1}=1/6$ per day, and $\mu =10^{-3}$ per day. We now derive plausible ranges for the other model parameters making use of the results from Ref. [48]. From Ref. [48, Equation (19)], we identify

$$R_{TT}=\sigma \nu_{ln}\left\langle k_{out}\right\rangle =\frac{\alpha_{1}\Phi_{T}}{\nu_{0}\nu_{1}}.$$

From Ref. [48] $\left\langle k_{out}\right\rangle =\frac{\Phi_{T}}{\nu }$, with $\left\langle k_{out}\right\rangle =2\times 10^{2}$, from which we obtain $\nu =10^{-2}$ per day, given the fixed value of $\Phi_{T}$. Ref. [48 also provides the following values: σ=0.1 and $\nu_{ln}=2.4\times 10^{-3}$ or $\nu_{ln}=2\times 10^{-2}$ depending on the disease [48, Table 2]. Thus, we derive $\alpha_{1}=\sigma \nu_{ln}\nu$, so that we conclude $\alpha_{1}=2.4\times 10^{-6}$ or $\alpha_{1}=2\times 10^{-5}$ per day per tick, respectively. From here, we assume values of $\alpha_{1}$ in the interval [10^−6^, 10^−4^].

We now turn to the transmission parameters (tick-to-host) $\beta_{1}$ and (host-to-tick) $\gamma_{1}$. From Ref. [48, Equations (6) and (7)], we have

$$R_{TH}=\sigma \nu_{hn}=\frac{\beta_{1}\Phi_{H}}{\mu_{0}\nu_{1}},\;R_{HT}=\nu_{lh}\left\langle k_{out}\right\rangle =\frac{\gamma_{1}\Phi_{T}}{\nu_{0}\left(\mu_{1}\;+\;\varphi_{1}\right)}.$$

Thus, we can write $\beta_{1}=\sigma \mu \nu \nu_{hn}∼10^{-6}$, from which we define a plausible range for $\beta_{1}$ as [10^−7^, 10^−5^]. Finally, $\gamma_{1}=\nu_{lh}\left(\mu +\varphi_{1}\right)$. As the value of $\nu_{lh}$ depends on the disease (it is either $\nu_{lh}=1.9\times 10^{-3}$ or $\nu_{lh}=7.3\times 10^{-2}$), we consider the interval [10^−5^, 10^−2^] for $\gamma_{1}$.

## Appendix B. Within-host invasion model

Following the same steps as those provided in Section 3.2, the next-generation matrix for the within-host invasion model is given by

$$K\equiv \left(\begin{array}{ll} b_{11} & b_{12}\\ b_{21} & b_{22} \end{array}\right),$$

where

$$b_{11}=\frac{\alpha_{2}{\bar{m}}_{0}}{\alpha_{1}\phi_{1|2}{\bar{m}}_{1}\;+\;\nu_{2}}+\frac{\alpha_{1}\phi_{1|2}{\bar{m}}_{1}}{\alpha_{1}\phi_{1|2}{\bar{m}}_{1}\;+\;\nu_{2}}\frac{\delta_{2}\left(1\;-\;\epsilon_{c}\right){\bar{m}}_{0}}{\nu_{c}},$$

$$b_{12}=\frac{\delta_{2}\left(1\;-\;\epsilon_{c}\right){\bar{m}}_{0}}{\nu_{c}},$$

$$b_{21}=\frac{\alpha_{2}\phi_{2|1}{\bar{m}}_{1}}{\alpha_{1}\phi_{1|2}{\bar{m}}_{1}\;+\;\nu_{2}}+\frac{\alpha_{1}\phi_{1|2}{\bar{m}}_{1}}{\alpha_{1}\phi_{1|2}{\bar{m}}_{1}\;+\;\nu_{2}}\frac{\left(\delta_{1}\;+\;\delta_{2}\right)\;\epsilon_{c}\;\left({\bar{m}}_{0}\;+\;\phi_{2|1}{\bar{m}}_{1}\right)\;+\;\delta_{2}\;\left(1\;-\;\epsilon_{c}\right)\phi_{2|1}{\bar{m}}_{1}}{\nu_{c}},$$

$$b_{22}=\frac{\left(\delta_{1}\;+\;\delta_{2}\right)\;\epsilon_{c}\;\left({\bar{m}}_{0}\;+\;\phi_{2|1}{\bar{m}}_{1}\right)\;+\;\delta_{2}\;\left(1\;-\;\epsilon_{c}\right)\phi_{2|1}{\bar{m}}_{1}}{\nu_{c}}.$$

When considering neutrality (*i.e.,* the invasive strain is the same as the resident strain), all infected tick populations ($m_{1}$, $m_{2}$, and $m_{c}$) are infected with the same viral strain. Thus, we have $\nu_{2}=\nu_{c}=\nu_{1}$ and $\alpha_{2}=\alpha_{1}$. We also set $\delta_{1}=\delta_{2}=\frac{\alpha_{1}}{2}$, representing that ticks in the $m_{c}$ compartment will transmit virus at the same overall rate as ticks in the $m_{1}$ compartment (i.e., $\delta_{1}+\delta_{2}=\alpha_{1}$). Furthermore, we would expect $\phi_{1|2}=\phi_{2|1}=0$, since in the within-host environment (a tick) the transmitted strain is likely to be rare compared to the established strain and if the resident strain is the same as the invasive strain, then both strains have the same within-host fitness, implying that the rare transmitted strain will have no within-host advantage over the established strain, and will be unable to establish co-infection in the host (tick). With these limits, the elements of the next-generation matrix become

$$b_{11}=\frac{\alpha_{1}{\bar{m}}_{0}}{\nu_{1}},$$

$$b_{12}=\frac{\alpha_{1}\left(1\;-\;\epsilon_{c}\right){\bar{m}}_{0}}{2\nu_{1}},$$

$$b_{21}=0,$$

$$b_{22}=\frac{\alpha_{1}\epsilon_{c}{\bar{m}}_{0}}{\nu_{1}}.$$

The eigenvalues are then $b_{11}$ and $b_{22}$, which are equal to 1 and $\epsilon_{c}$, respectively, since ${\bar{m}}_{0}=\frac{\nu_{1}}{\alpha_{1}}$. Thus, we can conclude that $R_{I}=1$, given $\epsilon_{c}\in [0,\;1]$.

## Appendix C. Alizon’s proposal

Alizon [37] proposed a model with doubly infected hosts (with the same viral strain), and which seemed a sufficient approach to achieve neutrality. We have considered a generalisation of the model originally proposed in Ref. [37], and which is described by the ODE system (13). By setting $\frac{dm_{0}}{\;dt}=\frac{dm_{1}}{\;dt}=\frac{dM_{1}}{\;dt}=0$, and $m_{2}=m_{c}=M_{2}=0$, one obtains the endemic equilibrium for the resident strain ${\tilde{E}}_{1}=\left({\tilde{m}}_{0},{\tilde{m}}_{1},\;0,\;0,{\tilde{M}}_{1},0\right)$. We then compute the invasion reproduction number of the invasive strain by considering the invasive sub-system, linearised around the resident strain endemic equilibrium, ${\tilde{E}}_{1}$:

$$\frac{dm_{2}}{\;dt}=-\nu_{2}m_{2}+{\tilde{m}}_{0}\lambda_{2}-m_{2}(\alpha_{1}{\tilde{m}}_{1}+2\;\kappa_{1}\;{\tilde{M}}_{1}),$$

$$\frac{dm_{c}}{\;dt}=-\nu_{c}m_{c}+{\tilde{m}}_{0}\left(\lambda_{1,c}+\lambda_{2,c}\right)+{\tilde{m}}_{1}\left(\lambda_{2}+\lambda_{2,c}+\Lambda_{2}\right)+m_{2}(\alpha_{1}{\tilde{m}}_{1}+2\;\kappa_{1}\;{\tilde{M}}_{1}),$$

$$\frac{dM_{2}}{dt}=-v_{2}M_{2}+{\tilde{m}}_{0}\Lambda_{2}.$$

The Jacobian matrix of the invasive sub-system is

$$\tilde{J}\equiv \left(\begin{array}{ccc} \begin{array}{c} -\nu_{2}+{\tilde{m}}_{0}\alpha_{2}\\ -(\alpha_{1}{\tilde{m}}_{1}+2\kappa_{1}{\tilde{M}}_{1}) \end{array} & {\tilde{m}}_{0}\delta_{2}(1-\epsilon_{c}) & 2{\tilde{m}}_{0}\kappa_{2}\left(1-\epsilon_{2}\right)\\ \begin{array}{c} {\tilde{m}}_{1}\alpha_{2}\\ +\alpha_{1}{\tilde{m}}_{1}+2\kappa_{1}{\tilde{M}}_{1} \end{array} & \begin{array}{c} -\nu_{c}+{\tilde{m}}_{0}\left(\delta_{1}+\delta_{2}\right)\epsilon_{c}\\ +{\tilde{m}}_{1}\delta_{2} \end{array} & 2{\tilde{m}}_{1}\kappa_{2}\\ 0 & 0 & -v_{2}+2{\tilde{m}}_{0}\kappa_{2}\epsilon_{2} \end{array}\right),$$

and can be decomposed as follows

$$\begin{array}{c} \tilde{T}\equiv \left(\begin{array}{ccc} {\tilde{m}}_{0}\alpha_{2} & {\tilde{m}}_{0}\delta_{2}\left(1-\epsilon_{c}\right) & 2\;{\tilde{m}}_{0}\kappa_{2}\left(1-\epsilon_{2}\right)\\ {\tilde{m}}_{1}\alpha_{2} & {\tilde{m}}_{0}\left(\delta_{1}+\delta_{2}\right)\;\epsilon_{c}+{\tilde{m}}_{1}\delta_{2} & 2\;{\tilde{m}}_{1}\kappa_{2}\\ 0 & 0 & 2\;{\tilde{m}}_{0}\kappa_{2}\epsilon_{2} \end{array}\right),\;\text{and}\\ \tilde{V}\equiv \left(\begin{array}{ccc} -\nu_{2}-(\alpha_{1}{\tilde{m}}_{1}+2\;\kappa_{1}{\tilde{M}}_{1}) & 0 & 0\\ \alpha_{1}{\tilde{m}}_{1}+2\;\kappa_{1}{\tilde{M}}_{1} & -\nu_{c} & 0\\ 0 & 0 & -v_{2} \end{array}\right). \end{array}$$

Finally, the next-generation matrix, $K\equiv \tilde{T}{\left[-\tilde{V}\right]}^{-1}$, is given by

$$K\equiv \left(\begin{array}{ccc} A & B & C\\ D & E & F\\ G & H & I \end{array}\right),$$

where

$$A=\frac{\alpha_{2}{\tilde{m}}_{0}}{\nu_{2}\;+\;\alpha_{1}{\tilde{m}}_{1}\;+\;2\kappa_{1}{\tilde{M}}_{1}}\;+\;\frac{\alpha_{1}{\tilde{m}}_{1}\;+\;2\kappa_{1}{\tilde{M}}_{1}}{\nu_{2}\;+\;\alpha_{1}{\tilde{m}}_{1}\;+\;2\kappa_{1}{\tilde{M}}_{1}}\frac{\delta_{2}\left(1\;-\;\epsilon_{c}\right){\tilde{m}}_{0}}{\nu_{c}},$$

$$B=\frac{\delta_{2}\left(1\;-\;\epsilon_{c}\right){\tilde{m}}_{0}}{\nu_{c}},$$

$$C=\frac{2\kappa_{2}\left(1\;-\;\epsilon_{2}\right){\tilde{m}}_{0}}{v_{2}},$$

$$D=\frac{\alpha_{2}{\tilde{m}}_{1}}{\nu_{2}\;+\;\alpha_{1}{\tilde{m}}_{1}\;+\;2\kappa_{1}{\tilde{M}}_{1}}\;+\;\frac{\alpha_{1}{\tilde{m}}_{1}\;+\;2\kappa_{1}{\tilde{M}}_{1}}{\nu_{2}\;+\;\alpha_{1}{\tilde{m}}_{1}\;+\;2\kappa_{1}{\tilde{M}}_{1}}\frac{\left(\delta_{1}\;+\;\delta_{2}\right)\;\epsilon_{c}{\tilde{m}}_{0}\;+\;\delta_{2}{\tilde{m}}_{1}}{\nu_{c}},$$

$$E=\frac{\left(\delta_{1}\;+\;\delta_{2}\right)\;\epsilon_{c}{\tilde{m}}_{0}\;+\;\delta_{2}{\tilde{m}}_{1}}{\nu_{c}},$$

$$F=\frac{2\kappa_{2}{\tilde{m}}_{1}}{v_{2}},$$

G=0,

H=0,

$$I=\frac{2\kappa_{2}\epsilon_{2}{\tilde{m}}_{0}}{v_{2}}.$$

The invasion reproduction number, $R_{I}$, is given by the largest eigenvalue of K. One eigenvalue is given by the matrix element I. The other eigenvalues are those of the sub-matrix

$$K_{2\times 2}\equiv \left(\begin{array}{cc} A & B\\ D & E \end{array}\right),$$

with the largest of the two given by

$$R_{I}=\frac{(A\;+\;E)\;+\;\sqrt{{\left(A\;+\;E\right)}^{2}\;-\;4(AE\;-\;BD)}}{2}.$$

### Appendix C.1. Proof of neutrality

When considering neutrality (*i.e.,* the invasive strain is the same as the resident strain), we set $\nu_{2}=\nu_{1}=\nu$, $v_{2}=v_{1}=v$, $\alpha_{2}=\alpha_{1}=\alpha$, $\delta_{2}=\kappa_{2}=\delta_{1}=\kappa_{1}=\kappa$, and $\epsilon_{c}=\epsilon_{2}=\epsilon_{1}=\epsilon$. Here we will show that under these neutrality conditions, $R_{I}=1$, for the particular case where $\nu_{0}=v=\nu$ and κ=α/2. We make the simplifying assumption that infection does not affect the death rates since infection with CCHFV is asymptomatic in its animal hosts. The assumption that κ=α/2 is realistic because we are considering viral infection. As Alizon mentioned in Ref. [37], if a host infected by a given strain is re-infected by the exact same strain, we do not expect to see a change in viral load (and hence in transmission rate); that is, a singly infected tick can become doubly infected with the same strain in this model, but becoming doubly infected does not affect its viral load. Under these conditions, the endemic equilibrium for the resident strain satisfies

(C.1)
$${\tilde{m}}_{0}\;+\;{\tilde{m}}_{1}\;+\;{\tilde{M}}_{1}=\frac{\Phi }{\nu },$$

with

(C.2)
$${\tilde{m}}_{0}=\frac{\nu }{\alpha },$$

(C.3)
$$\begin{array}{ll} {\tilde{m}}_{1} & \;=\frac{{\tilde{m}}_{0}(1\;-\;\epsilon )\left(\alpha \Phi \;-\;\nu^{2}\right)}{\alpha \Phi \;-\;\nu^{2}\epsilon }. \end{array}$$

Hence, we have

(C.4)
$$\nu \;+\;\alpha ({\tilde{m}}_{1}\;+\;{\tilde{M}}_{1})=\frac{\alpha \Phi }{\nu }.$$

Making use of Eqs. (C.1), (C.2), and (C.4), the relevant elements of the next-generation matrix simplify to

$$\begin{array}{l} A\;=\frac{\alpha {\tilde{m}}_{0}}{\nu \;+\;\alpha ({\tilde{m}}_{1}\;+\;{\tilde{M}}_{1})}\;+\;\frac{\alpha ({\tilde{m}}_{1}\;+\;{\tilde{M}}_{1})}{\nu \;+\;\alpha ({\tilde{m}}_{1}\;+\;{\tilde{M}}_{1})}\frac{\alpha (1\;-\;\epsilon ){\tilde{m}}_{0}}{2\nu },\\ \;=\frac{1}{\Phi }\left[\nu {\tilde{m}}_{0}\;+\;\frac{1}{2}\nu (1-\epsilon )\left(\frac{\Phi }{\nu }-{\tilde{m}}_{0}\right)\right], \end{array}$$

$$B=\frac{\alpha (1\;-\;\epsilon ){\tilde{m}}_{0}}{2\nu }=\frac{1}{2}(1-\epsilon ),$$

$$\begin{array}{l} D\;=\frac{\alpha {\tilde{m}}_{1}}{\nu \;+\;\alpha ({\tilde{m}}_{1}\;+\;{\tilde{M}}_{1})}\;+\;\frac{\alpha ({\tilde{m}}_{1}\;+\;{\tilde{M}}_{1})}{\nu \;+\;\alpha ({\tilde{m}}_{1}\;+\;{\tilde{M}}_{1})}\frac{\alpha \epsilon {\tilde{m}}_{0}\;+\;\frac{1}{2}\alpha {\tilde{m}}_{1}}{\nu },\\ \;=\frac{1}{\Phi }\left[\nu {\tilde{m}}_{1}\;+\;\nu \left(\epsilon \;+\;\frac{\alpha }{2\nu }{\tilde{m}}_{1}\right)\left(\frac{\Phi }{\nu }-{\tilde{m}}_{0}\right)\right], \end{array}$$

$$E=\frac{\alpha \epsilon {\tilde{m}}_{0}\;+\;\frac{1}{2}\alpha {\tilde{m}}_{1}}{\nu }=\epsilon \;+\;\frac{\alpha }{2\nu }{\tilde{m}}_{1},$$

$$I=\frac{\alpha \epsilon {\tilde{m}}_{0}}{\nu }=\epsilon .$$

Since I=ϵ≤1, we need to show that

$$R_{I}=\frac{(A\;+\;E)\;+\;\sqrt{{\left(A\;+\;E\right)}^{2}\;-\;4\;(AE\;-\;BD)}}{2}=1.$$

It can be shown that this is true if and only if

(C.5)
A+E−(AE−BD)=1.

Thus, in order to show that $R_{I}=1$, it is sufficient to show that Eq. (C.5) holds. We have,

$$AE-BD=\frac{1}{\Phi }\epsilon \nu \left({\tilde{m}}_{0}\;+\;\frac{1}{2}{\tilde{m}}_{1}\right).$$

Thus, we can write

$$\begin{array}{l} A\;+\;E-(AE-BD)\;=\frac{1}{\Phi }\left[\nu {\tilde{m}}_{0}\;+\;\frac{1}{2}\nu (1-\epsilon )\left(\frac{\Phi }{\nu }-{\tilde{m}}_{0}\right)\;+\;\Phi \epsilon \;+\;\frac{\alpha \Phi }{2\nu }{\tilde{m}}_{1}-\epsilon \nu \left({\tilde{m}}_{0}\;+\;\frac{1}{2}{\tilde{m}}_{1}\right)\right],\\ \;=\frac{1}{\Phi }\left[\frac{1}{2}\nu {\tilde{m}}_{0}(1-\epsilon )\;+\;\frac{\Phi }{2}(1\;+\;\epsilon )\;+\;\frac{1}{2\nu }{\tilde{m}}_{1}(\alpha \Phi -\nu^{2}\epsilon )\right]. \end{array}$$

Substituting ${\tilde{m}}_{1}$ with its expression from Eq. (C.3) gives

$$A\;+\;E-\left(AE-BD\right)=\frac{1}{\Phi }\left[\frac{\alpha \Phi }{2\nu }{\tilde{m}}_{0}\left(1-\epsilon \right)\;+\;\frac{\Phi }{2}\left(1\;+\;\epsilon \right)\right].$$

Finally, by substituting Eq. (C.2) in the previous equation, we have

A+E−(AE−BD)=1.

## Appendix D. The two-slot model of co-infection and co-transmission

### Appendix D.1. Transmission events

We list here the transmission events which lead to the two-slot mathematical model introduced in Eq. (15) grouped by the type of transmission. $T_{0}$ denotes a susceptible tick, $T_{1}$ and $T_{2}$ denote a singly infected tick with $V_{1}$ and $V_{2}$, respectively, $T_{11}$ and $T_{22}$ denote a doubly infected tick with $V_{1}$ and $V_{2}$, respectively, and $T_{c}$ denotes a co-infected tick (with $V_{1}$ and $V_{2}$).

- Transmission from a singly infected tick to a susceptible tick:
  $T_{0}\;+\;T_{1}\to T_{1}\;+\;T_{1}$ with rate $\alpha_{1}m_{0}m_{1}$,
  $T_{0}\;+\;T_{2}\to T_{2}\;+\;T_{2}$ with rate $\alpha_{2}m_{0}m_{2}$.
- Transmission from a singly infected tick to a singly infected tick:
  $T_{1}\;+\;T_{1}\to T_{11}\;+\;T_{1}$ with rate $\alpha_{1}m_{1}m_{1}$,
  $T_{1}\;+\;T_{2}\to T_{c}\;+\;T_{2}$ with rate $\alpha_{2}m_{1}m_{2}$,
  $T_{2}\;+\;T_{1}\to T_{c}\;+\;T_{1}$ with rate $\alpha_{1}m_{2}m_{1}$,
  $T_{2}\;+\;T_{2}\to T_{22}\;+\;T_{2}$ with rate $\alpha_{2}m_{2}m_{2}$.
- Transmission from a doubly infected tick to a susceptible tick:
  $T_{0}\;+\;T_{11}\to T_{1}\;+\;T_{11}$ with rate $\left(1-\epsilon_{1}\right)2\kappa_{1}m_{0}M_{1}$,
  $T_{0}\;+\;T_{11}\to T_{11}\;+\;T_{11}$ with rate $\epsilon_{1}2\kappa_{1}m_{0}M_{1}$.
  $T_{0}\;+\;T_{22}\to T_{2}\;+\;T_{22}$ with rate $\left(1-\epsilon_{2}\right)2\kappa_{2}m_{0}M_{2}$,
  $T_{0}\;+\;T_{22}\to T_{22}\;+\;T_{22}$ with rate $\epsilon_{2}2\kappa_{2}m_{0}M_{2}$.
- Transmission from a co-infected tick to a susceptible tick:
  $T_{0}\;+\;T_{c}\to T_{1}\;+\;T_{c}$ with rate $\left(1-\epsilon_{c}\right)\delta_{1}m_{0}m_{c}$,
  $T_{0}\;+\;T_{c}\to T_{2}\;+\;T_{c}$ with rate $\left(1-\epsilon_{c}\right)\delta_{2}m_{0}m_{c}$,
  $T_{0}\;+\;T_{c}\to T_{c}\;+\;T_{c}$ with rate $\frac{2\delta_{1}\delta_{2}}{\delta_{1}\;+\;\delta_{2}}\epsilon_{c}m_{0}m_{c}$,
  $T_{0}\;+\;T_{c}\to T_{11}\;+\;T_{c}$ with rate $\frac{\delta_{1}^{2}}{\delta_{1}\;+\;\delta_{2}}\epsilon_{c}m_{0}m_{c}$,
  $T_{0}\;+\;T_{c}\to T_{22}\;+\;T_{c}$ with rate $\frac{\delta_{2}^{2}}{\delta_{1}\;+\;\delta_{2}}\epsilon_{c}m_{0}m_{c}$.
- Transmission from a co-infected tick to a singly infected tick:
  $T_{1}\;+\;T_{c}\to T_{11}\;+\;T_{c}$ with rate $\delta_{1}m_{1}m_{c}$,
  $T_{1}\;+\;T_{c}\to T_{c}\;+\;T_{c}$ with rate $\delta_{2}m_{1}m_{c}$.
  $T_{2}\;+\;T_{c}\to T_{22}\;+\;T_{c}$ with rate $\delta_{2}m_{2}m_{c}$,
  $T_{2}\;+\;T_{c}\to T_{c}\;+\;T_{c}$ with rate $\delta_{1}m_{2}m_{c}$.
- Transmission from a doubly infected tick to a singly infected tick:
  $T_{1}\;+\;T_{11}\to T_{11}\;+\;T_{11}$ with rate $2\kappa_{1}m_{1}M_{1}$,
  $T_{2}\;+\;T_{22}\to T_{22}\;+\;T_{22}$ with rate $2\kappa_{2}m_{2}M_{2}$,
  $T_{1}\;+\;T_{22}\to T_{c}\;+\;T_{22}$ with rate $2\kappa_{2}m_{1}M_{2}$,
  $T_{2}\;+\;T_{11}\to T_{c}\;+\;T_{11}$ with rate $2\kappa_{1}m_{2}M_{1}$.

### Appendix D.2. Existence of the endemic equilibrium of $V_{1}$

In Appendix C.1 we have shown the endemic equilibrium (EE) of $V_{1}$ can be written as ${\tilde{E}}_{1}=({\tilde{m}}_{0},{\tilde{m}}_{1},\;0,\;0,\;{\tilde{M}}_{1},\;0)$. We have also shown the neutrality of the invasion reproduction number in Alizon’s model under the assumption κ=α/2. This assumption simplifies the next-generation matrix and helps to prove neutrality. Our two-slot model, as an extension of Alizon’s model, shares the same resident strain EE. In this section, we will discuss the existence of the resident strain EE when κ≠α/2, referring to Appendix C.1 for the case κ=α/2. We, thus, write the EE as $E_{1}^{\prime }=\left(m_{0}^{\prime },m_{1}^{\prime },\;0,\;0,\;M_{1}^{\prime },0\right)$. We then compute the invasion reproduction number $R_{1}^{\prime }$ and prove the neutrality in Appendix D.3 and Appendix D.4, respectively.

First, we consider the resident sub-system, where $m_{2}=m_{C}=M_{2}=0$ and assume $\nu_{0}=\nu_{1}=\nu_{2}=\nu_{c}=v_{1}=v_{2}=\nu$, without loss of generality. We have

(D.1)
$$\begin{array}{ll} \frac{dm_{0}}{\;dt} & \;=\Phi -\nu m_{0}-\left(\alpha_{1}m_{1}\;+\;2\;\kappa_{1}M_{1}\right)m_{0},\\ \frac{dm_{1}}{\;dt} & \;=-\nu m_{1}-\left(\alpha_{1}m_{1}\;+\;2\;\kappa_{1}M_{1}\right)m_{1}\;+\;\left(\alpha_{1}m_{1}\;+\;2\;\kappa_{1}\left(1-\epsilon_{1}\right)M_{1}\right)m_{0},\\ \frac{dM_{1}}{\;dt} & \;=-\nu M_{1}\;+\;\left(\alpha_{1}m_{1}\;+\;2\;\kappa_{1}M_{1}\right)m_{1}\;+\;2\;\epsilon_{1}\kappa_{1}M_{1}m_{0}. \end{array}$$

We compute the basic reproduction number of this sub-system at the VFE

$E_{0}=\left(m_{0}^{⋆},0,0,0,0,0\right)$, where $m_{0}^{⋆}=\frac{\Phi }{\nu }$. We can write

(D.2)
$$R_{1}^{\prime }=max\left\{\frac{\Phi \alpha_{1}}{\nu^{2}},\frac{2\Phi \epsilon_{1}\;\kappa_{1}}{\nu^{2}}\right\}.$$

By setting $\frac{d\left(m_{1}\;+\;M_{1}\right)}{dt}=0$ and $\frac{dm_{0}}{\;dt}=0$, we obtain the following equations:

(D.3)
$$\alpha_{1}m_{1}^{\prime }\;+\;2\kappa_{1}M_{1}^{\prime }=\frac{\left(m_{1}^{\prime }\;+\;M_{1}^{\prime }\right)\;\nu }{m_{0}^{\prime }},$$

(D.4)
$$m_{1}^{\prime }\;+\;M_{1}^{\prime }=\frac{\Phi }{\nu }-m_{0}^{\prime }.$$

From Eq. (D.4), we have $m_{0}^{\prime }<\frac{\Phi }{\nu }$ to ensure positive $m_{1}^{\prime }$ and $M_{1}^{\prime }$. By combining Eqs. (D.3) and (D.4), we then get

(D.5)
$$\alpha_{1}m_{1}^{\prime }\;+\;2\;\kappa_{1}M_{1}^{\prime }=\frac{\Phi }{m_{0}^{\prime }}-\nu .$$

By solving Eq. (D.4) and Eq. (D.5), one obtains

(D.6)
$$m_{1}^{\prime }=\frac{\Phi \;-\;\nu m_{0}^{\prime }}{2\kappa_{1}\;-\;\alpha_{1}}\left(\frac{1}{m_{0}^{\prime }}-\frac{\alpha_{1}}{\nu }\right),\;\text{and}\;\;M_{1}^{\prime }=\frac{\Phi \;-\;\nu m_{0}^{\prime }}{\alpha_{1}\;-\;2\kappa_{1}}\left(\frac{1}{m_{0}^{\prime }}-\frac{2\kappa_{1}}{\nu }\right).$$

We conclude that to ensure positive values for $m_{1}^{\prime }$ and $M_{1}^{\prime }$, we require the following conditions:

- if $\alpha_{1}>2\kappa_{1}$, $\frac{\nu }{\alpha_{1}}<m_{0}^{\prime }<\frac{\nu }{2\kappa_{1}}$, or
- if $\alpha_{1}<2\kappa_{1}$, $\frac{\nu }{2\kappa_{1}}<m_{0}^{\prime }<\frac{\nu }{\alpha_{1}}$.

If $2\kappa_{1}=\alpha_{1}$, we refer to Appendix C.1. Substituting Eqs. (D.5) and (D.6) into $\frac{dM_{1}}{\;dt}=0$, we derive the following cubic equation for $m_{0}^{\prime }$:

$$Q\left(m_{0}\right)=A_{3}m_{0}^{3}\;+\;A_{2}m_{0}^{2}\;+\;A_{1}m_{0}\;+\;A_{0}=0,$$

where $A_{3}=-2\kappa_{1}\epsilon_{1}\alpha_{1}$, $A_{2}=2\kappa_{1}\epsilon_{1}\nu -2\kappa_{1}\nu \;+\;\alpha_{1}\nu$, $A_{1}=2\Phi \kappa_{1}$, and $A_{0}=-\Phi \nu$. It is easy to observe that $A_{3}<0,Q(0)<0$, and $Q^{\prime }(0)>0$. We therefore have one negative root for $Q\left(m_{0}^{\prime }\right)=0$ and two critical points (i.e., where $Q^{\prime }\left(m_{0}^{\prime }\right)=0$) distributed at different sides of the y-axis. From Eqs. (D.4) and (D.6), we have three important values for $m_{0}^{\prime }:\frac{\Phi }{\nu },\frac{\nu }{\alpha_{1}}$, and $\frac{\nu }{2\kappa_{1}}$.

We then have the following values:

$$Q\left(\frac{\Phi }{\nu }\right)=\Phi \nu \left(1-\frac{\Phi }{\nu }\frac{2\kappa_{1}\epsilon_{1}}{\nu }\right)\left(\frac{\Phi }{\nu }\frac{\alpha_{1}}{\nu }-1\right),$$

$$Q\left(\frac{\nu }{\alpha_{1}}\right)=\nu^{2}\left(\frac{\Phi }{\nu }-\frac{\nu }{\alpha_{1}}\right)\left(\frac{2\kappa_{1}}{\alpha_{1}}-1\right),$$

$$Q\left(\frac{\nu }{2\kappa_{1}}\right)=\frac{\nu^{3}}{2\kappa_{1}}\left(\frac{\alpha_{1}}{2\kappa_{1}}-1\right)\left(1-\epsilon_{1}\right).$$

We can now discuss the existence of a real and positive $m_{0}^{\prime }$, when $R_{1}^{\prime }>1$ and $0\leq \epsilon_{1}\leq 1$. We need to consider the following cases:

- when $\frac{2\;\kappa_{1}\;\epsilon_{1}\;\Phi }{\nu^{2}}\leq 1<\frac{\alpha_{1}\Phi }{\nu^{2}}$, then $R_{1}^{\prime }=\frac{\alpha_{1}\Phi }{\nu^{2}}$, $Q\left(\frac{\Phi }{\nu }\right)\geq 0$ and $\frac{\nu }{\alpha_{1}}<\frac{\Phi }{\nu }$, $\frac{\nu }{2\kappa_{1}}\geq \epsilon_{1}\frac{\Phi }{\nu }$. We need to consider two separate cases:
  - if $\alpha_{1}>2\kappa_{1}$, that is, $\frac{\nu }{\alpha_{1}}<m_{0}^{\prime }<\frac{\nu }{2\kappa_{1}}$, then $Q\left(\frac{\nu }{\alpha_{1}}\right)<0$, and $Q\left(\frac{\nu }{2\kappa_{1}}\right)\geq 0$. We thus have a unique solution for $Q\left(m_{0}^{\prime }\right)=0$ on $\left(\frac{\nu }{\alpha_{1}},min\left\{\frac{\nu }{2\kappa_{1}},\frac{\Phi }{\nu }\right\}\right]$.
  - if $\alpha_{1}<2\kappa_{1}$, that is, $\frac{\nu }{2\kappa_{1}}<m_{0}^{\prime }<\frac{\nu }{\alpha_{1}}$ and $2\kappa_{1}\epsilon_{1}<\alpha_{1}<2\kappa_{1}$, then $Q\left(\frac{\nu }{\alpha_{1}}\right)>0$ and $Q\left(\frac{\nu }{2\kappa_{1}}\right)\leq 0$. We thus have a unique solution on $\left[\frac{\nu }{2\kappa_{1}},\frac{\nu }{\alpha_{1}}\right)$.
- when $\frac{\alpha_{1}\Phi }{\nu^{2}}\leq 1<\frac{2\kappa_{1}\epsilon_{1}\Phi }{\nu^{2}}$, then $R_{1}^{\prime }=\frac{2\kappa_{1}\epsilon_{1}\Phi }{\nu^{2}}$, $\alpha_{1}<2\kappa_{1}\epsilon_{1}<2\kappa_{1}$, $\frac{\nu }{2\kappa_{1}}<\epsilon_{1}\frac{\Phi }{\nu }$, and $\frac{\nu }{\alpha_{1}}\geq \frac{\Phi }{\nu }$, since $\alpha_{1}<2\kappa_{1}$; that is, $\frac{\nu }{2\kappa_{1}}<m_{0}^{\prime }<\frac{\nu }{\alpha_{1}}$. Then we can further constrain the solution to $\frac{\nu }{2\kappa_{1}}<m_{0}^{\prime }<\frac{\Phi }{\nu }$, and we have $Q\left(\frac{\nu }{2\kappa_{1}}\right)\leq 0$, and $Q\left(\frac{\Phi }{\nu }\right)>0$. Thus a unique solution can be found on $\left[\frac{\nu }{2\kappa_{1}},\frac{\Phi }{\nu }\right)$.
- when $\frac{\alpha_{1}\Phi }{\nu^{2}}>1$ and $\frac{2\kappa_{1}\epsilon_{1}\Phi }{\nu^{2}}>1$, then $\frac{\nu }{\alpha_{1}}<\frac{\Phi }{\nu }$, $\frac{\nu }{2\kappa_{1}}<\epsilon_{1}\frac{\Phi }{\nu }$, and $Q\left(\frac{\Phi }{\nu }\right)<0$. We need to consider two different cases:
  - if $\alpha_{1}>2\kappa_{1}$, that is $\frac{\nu }{\alpha_{1}}<m_{0}^{\prime }<\frac{\nu }{2\kappa_{1}}$, then $Q\left(\frac{\nu }{\alpha_{1}}\right)<0$ and $Q\left(\frac{\nu }{2\kappa_{1}}\right)\geq 0$. We thus have a unique solution on $\left(\frac{\nu }{\alpha_{1}},\frac{\nu }{2\kappa_{1}}\right]$.
  - if $\alpha_{1}<2\kappa_{1}$, that is $\frac{\nu }{2\kappa_{1}}<m_{0}^{\prime }<\frac{\nu }{\alpha_{1}}$, then $Q\left(\frac{\nu }{\alpha_{1}}\right)>0$ and $Q\left(\frac{\nu }{2\kappa_{1}}\right)\leq 0$. We thus have a unique solution on $\left[\frac{\nu }{2\kappa_{1}},\frac{\nu }{\alpha_{1}}\right)$.

Therefore, when $R_{1}^{\prime }>1$ and $0\leq \epsilon_{1}\leq 1$, a unique real and non-negative solution of $E_{1}^{\prime }=\left(m_{0}^{\prime },\;m_{1}^{\prime },0,\;0,\;M_{1}^{\prime },\;0\right)$ is guaranteed. We can ensure three real roots (i.e., one negative and two positive roots) for $Q\left(m_{0}\right)=0$, such that we identify the value of $m_{0}^{\prime }$ which is real and positive, with $m_{1}^{\prime }$ and $M_{1}^{\prime }$ real and positive as well. To do so we make use of the general formula for a cubic equation (with $A_{3}\neq 0$) [56]:

(D.7)
$$m_{0}^{\prime }=-\frac{1}{3A_{3}}\left(A_{2}\;+\;\Delta \;+\;\frac{\Delta_{0}}{\Delta }\right),$$

with

$$\begin{array}{ll} \Delta_{0} & \;=A_{2}^{2}-3A_{1}A_{3}\\ \Delta_{1} & \;=2A_{2}^{3}-9A_{1}A_{2}A_{3}\;+\;27A_{0}A_{3}^{2}\\ \Delta & \;={\left(\frac{-1\;+\;\sqrt{-3}}{2}\right)}^{2}{\left(\frac{\Delta_{1}\;+\;\sqrt{\Delta_{1}^{2}\;-\;4\Delta_{0}^{3}}}{2}\right)}^{1/3}. \end{array}$$

We note that $m_{0}^{\prime }$ is real even though Δ is a complex number. Another expression for the solution of $m_{0}^{\prime }$ making use of trigonometric functions can be found in Ref. [57]

### Appendix D.3. Invasion reproduction number

We can now compute the invasion reproduction number, $R_{I}^{\prime }$, of $V_{2}$ for the invasion sub-system, linearised around the endemic equilibrium $E_{1}^{\prime }$. We have the following Jacobian matrix:

$$J^{\prime }\equiv \left(\begin{array}{ccc} \begin{array}{c} -\nu -\alpha_{1}m_{1}^{\prime }\\ +\alpha_{2}m_{0}^{\prime }-2\kappa_{1}M_{1}^{\prime } \end{array} & \delta_{2}\left(1-\epsilon_{c}\right)m_{0}^{\prime } & 2\kappa_{2}\left(1-\epsilon_{2}\right)m_{0}^{\prime }\\ \begin{array}{c} \left(\alpha_{1}+\alpha_{2}\right)m_{1}^{\prime }\\ +2\kappa_{1}M_{1}^{\prime } \end{array} & -\nu +\delta_{2}\left(\frac{2\delta_{1}\epsilon_{c}}{\delta_{1}+\delta_{2}}m_{0}^{\prime }+m_{1}^{\prime }\right) & 2\kappa_{2}m_{1}^{\prime }\\ 0 & \frac{\delta_{2}^{2}\epsilon_{c}}{\delta_{1}+\delta_{2}}m_{0}^{\prime } & -\nu +2\;\epsilon_{2}\;\kappa_{2}\;m_{0}^{\prime } \end{array}\right),$$

which can be decomposed as follows

$$T^{\prime }\equiv \left(\begin{array}{ccc} \alpha_{2}m_{0}^{\prime } & \delta_{2}\left(1-\epsilon_{c}\right)m_{0}^{\prime } & 2\kappa_{2}\left(1-\epsilon_{2}\right)m_{0}^{\prime }\\ \alpha_{2}m_{1}^{\prime } & \delta_{2}\left(\frac{2\delta_{1}\epsilon_{c}}{\delta_{1}+\delta_{2}}m_{0}^{\prime }+m_{1}^{\prime }\right) & 2\kappa_{2}m_{1}^{\prime }\\ 0 & \frac{\delta_{2}^{2}\epsilon_{c}}{\delta_{1}+\delta_{2}}m_{0}^{\prime } & 2\epsilon_{2}\kappa_{2}m_{0}^{\prime } \end{array}\right),$$

and

$$V^{\prime }\equiv \left(\begin{array}{ccc} -\nu -\alpha_{1}m_{1}^{\prime }-2\kappa_{1}M_{1}^{\prime } & 0 & 0\\ \alpha_{1}m_{1}^{\prime }\;+\;2\kappa_{1}M_{1}^{\prime } & -\nu & 0\\ 0 & 0 & -\nu \end{array}\right).$$

We can compute the next-generation matrix, $K^{\prime }=T^{\prime }{\left[-V^{\prime }\right]}^{-1}$, given by:

$$K^{\prime }\equiv \left(\begin{array}{lll} d_{11} & d_{12} & d_{13}\\ d_{21} & d_{22} & d_{23}\\ d_{31} & d_{32} & d_{33} \end{array}\right),$$

where

$$d_{11}=\frac{\alpha_{2}m_{0}^{\prime }\;+\;\delta_{2}\left(1\;-\;\epsilon_{c}\right)\left(m_{1}^{\prime }\;+\;M_{1}^{\prime }\right)}{\Phi }m_{0}^{\prime },$$

$$d_{12}=\frac{\delta_{2}}{\nu }\left(1-\epsilon_{c}\right)m_{0}^{\prime },$$

$$d_{13}=\frac{2\kappa_{2}}{\nu }\left(1-\epsilon_{2}\right)m_{0}^{\prime },$$

$$d_{21}=\frac{\alpha_{2}}{\Phi }m_{0}^{\prime }m_{1}^{\prime }\;+\;\frac{\delta_{2}}{\Phi }\left(\frac{2\delta_{1}\epsilon_{c}}{\delta_{1}\;+\;\delta_{2}}m_{0}^{\prime }\;+\;m_{1}^{\prime }\right)\left(m_{1}^{\prime }\;+\;M_{1}^{\prime }\right),$$

$$d_{22}=\frac{\delta_{2}}{\nu }\left(\frac{2\epsilon_{c}\delta_{1}}{\delta_{1}\;+\;\delta_{2}}m_{0}^{\prime }\;+\;m_{1}^{\prime }\right),$$

$$d_{23}=\frac{2\kappa_{2}}{\nu }m_{1}^{\prime },$$

$$d_{31}=\frac{\delta_{2}}{\Phi }\frac{\epsilon_{c}\delta_{2}}{\delta_{1}\;+\;\delta_{2}}m_{0}^{\prime }\left(m_{1}^{\prime }\;+\;M_{1}^{\prime }\right),$$

$$d_{32}=\frac{\delta_{2}}{\nu }\frac{\epsilon_{c}\delta_{2}}{\delta_{1}\;+\;\delta_{2}}m_{0}^{\prime },$$

$$d_{33}=\frac{2\;\epsilon_{2}\;\kappa_{2}}{\nu }m_{0}^{\prime }.$$

We can obtain the invasion reproduction number, $R_{I}^{\prime }$, as a function of $m_{0}^{\prime }$ by substituting Eq. (D.6) into $K^{\prime }$ and computing its largest eigenvalue.

### Appendix D.4. Proof of neutrality

We consider the following limits: α1,α2→α,κ1,κ2,δ1,δ2→κ,andϵ1,ϵ2,ϵc→ϵ. In this limit, we can write the invasion reproduction number, $R_{I}^{\prime }$, at neutrality as follows:

(D.8)
$$\begin{array}{l} R_{I}^{\prime }=\frac{m_{0}^{\prime }}{2\;\nu \Phi \left(\Phi \;-\;\alpha \epsilon {m_{0}^{\prime }}^{2}\right)}\{\\ 2\Phi^{2}\kappa \;+\;\Phi \nu (\alpha -2\kappa (1-\epsilon ))m_{0}^{\prime }\\ \;-\Phi \alpha \epsilon \kappa (1\;+\;\epsilon ){m_{0}^{\prime }}^{2}-\alpha \epsilon \nu (\alpha -\kappa (1-\epsilon )){m_{0}^{\prime }}^{3}\\ \;\;+\;\left[4\Phi \alpha \epsilon \kappa \nu m_{0}^{\prime }\left(\Phi -\alpha \epsilon {m_{0}^{\prime }}^{2}\right)\left(\Phi (-3\;+\;\epsilon )\;+\;m_{0}^{\prime }\left(\nu (1-\epsilon )\;+\;2\alpha \epsilon m_{0}^{\prime }\right)\right)\right.\\ \;\;+\;\left(2\Phi^{2}\kappa \;+\;m_{0}^{\prime }(\Phi \nu (\alpha -2\kappa (1-\epsilon ))\right.\\ \left.{\left.{\left.\;-\epsilon \alpha m_{0}^{\prime }\left(\left(1\;+\;\epsilon )\Phi \kappa \;+\;\nu m_{0}^{\prime }(\alpha -(1-\epsilon )\kappa \right)\right)\right)}^{2}\right]}^{1/2}\right\}. \end{array}$$

Now we can prove the neutrality of $R_{I}^{\prime }$ in two scenarios:

1. when κ=α/2, the expression for $R_{I}^{\prime }$ reduces to
  $$\begin{array}{l} R_{I}^{\prime }=\frac{\alpha m_{0}^{\prime }\left(2\Phi \left(\Phi \;+\;\epsilon \nu m_{0}^{\prime }\right)\;-\;\epsilon \alpha (1\;+\;\epsilon )\left(\Phi \;+\;\nu m_{0}^{\prime }\right)m_{0}^{\prime 2}\right)}{4\Phi \nu \left(\Phi \;-\;\epsilon \alpha m_{0}^{2}\right)}\\ \;\;+\;\alpha m_{0}^{\prime }\left[\frac{\epsilon m_{0}^{\prime }\left(\left(2\epsilon \alpha m_{0}^{\prime }\;+\;(1\;-\;\epsilon )\nu \right)m_{0}^{\prime }\;-\;(3\;-\;\epsilon )\Phi \right)}{2\nu \Phi \left(\Phi \;-\;\epsilon \alpha m_{0}^{2}\right)}\right.\\ {\left.\;+\;{\left(\frac{2\Phi^{2}\;+\;\epsilon m_{0}^{\prime }\left(2\Phi \nu \;-\;\alpha m_{0}^{\prime }(1\;+\;\epsilon )\left(\Phi \;+\;\nu m_{0}^{\prime }\right)\right)}{4\Phi \nu \left(\Phi \;-\;\epsilon \alpha m_{0}^{\prime 2}\right)}\right)}^{2}\right]}^{1/2}. \end{array}$$
  By substituting $m_{0}^{\prime }={\tilde{m}}_{0}=\nu /\alpha$ as in Eq. (C.2), we obtain $R_{I}^{\prime }\equiv 1$, as desired.
2. when κ≠α/2, the expression for $R_{I}^{\prime }$ cannot be easily simplified. In this case, we perform a numerical study, making use of Mathematica to prove neutrality, which requires the expression of $m_{0}^{\prime }$ from Eq. (D.7).
